# Timing of complementary food introduction is not associated with inflammatory bowel disease risk: A prospective birth cohort study

**DOI:** 10.1093/ibd/izag093

**Published:** 2026-06-09

**Authors:** Ida Sigvardsson, Johnny Ludvigsson, Tereza Lerchova, Henrik Imberg, Ketil Størdal, Karl Mårild

**Affiliations:** Department of Pediatrics, Institute of Clinical Sciences, Sahlgrenska Academy, University of Gothenburg, Box 426, SE-405 30, Gothenburg, Sweden; Crown Princess Victoria Children’s Hospital, Linköping, Region Östergötland, Sweden; Division of Pediatrics, Department of Biomedical and Clinical Sciences, Linköping University, Linköping, Sweden; Department of Pediatrics, Institute of Clinical Sciences, Sahlgrenska Academy, University of Gothenburg, Box 426, SE-405 30, Gothenburg, Sweden; Statistiska Konsultgruppen Sweden, Gothenburg, Sweden; Department of Molecular and Clinical Medicine, Institute of Medicine, Sahlgrenska Academy, University of Gothenburg, Gothenburg, Sweden; Department of Pediatric Research, Faculty of Medicine, University of Oslo, Oslo, Norway; Children’s Center, Oslo University Hospital, Oslo, Norway; Department of Pediatrics, Institute of Clinical Sciences, Sahlgrenska Academy, University of Gothenburg, Box 426, SE-405 30, Gothenburg, Sweden; Department of Pediatrics, Queen Silvia Children’s Hospital, Gothenburg, Sweden

**Keywords:** inflammatory bowel disease, complementary feeding, food introduction, ABIS, MoBa

## Abstract

**Background:**

Complementary feeding (ie, food introduction besides formula or breast milk) imprints on the developing gut microbiome and immune system, which may have durable influences on disease risk. This study aimed to prospectively assess the association between the timing of complementary feeding and subsequent inflammatory bowel disease (IBD) risk.

**Methods:**

We followed 94 238 participants from the All Babies in Southeast Sweden (ABIS) (n = 11 947) and the Norwegian Mother, Father and Child (MoBa) (n = 82 291) cohorts from birth (1997-2009) through 2023 (mean age 16.5 [MoBa] to 25.2 [ABIS] years). National patient registers identified IBD diagnoses. The timing of complementary food introduction (<4, 4-5, or ≥6 months) was assessed using early-life food diaries and questionnaires. Latent class analyses identified 4 patterns across introductions of major food groups (eg, cereals and dairy). Cox regression estimated hazard ratios (aHRs) for IBD adjusted for socio-demographics and parental IBD. Sensitivity analysis additionally adjusted for breastfeeding duration, formula feeding, and perinatal factors.

**Results:**

Over 1 562 350 person-years of follow-up, 400 participants developed IBD (ABIS, n = 124; MoBa, n = 276). Overall timing of complementary food introduction was not associated with IBD (<4 months: aHR, 1.04 [95% CI, 0.67-1.60]; 4-5 months: aHR, 0.83 [95% CI, 0.63-1.10] vs ≥6 months). Also, aHRs for IBD by latent class analyses–defined introduction patterns approached 1. Results were consistent across cohorts, sensitivity analyses, and Crohn’s disease and ulcerative colitis subtypes.

**Conclusions:**

The findings from this binational birth cohort study indicate that neither the timing nor the pattern of complementary food introduction is a major risk factor for later development of IBD.

## Graphical Abstract

**Graphical Abstract:**
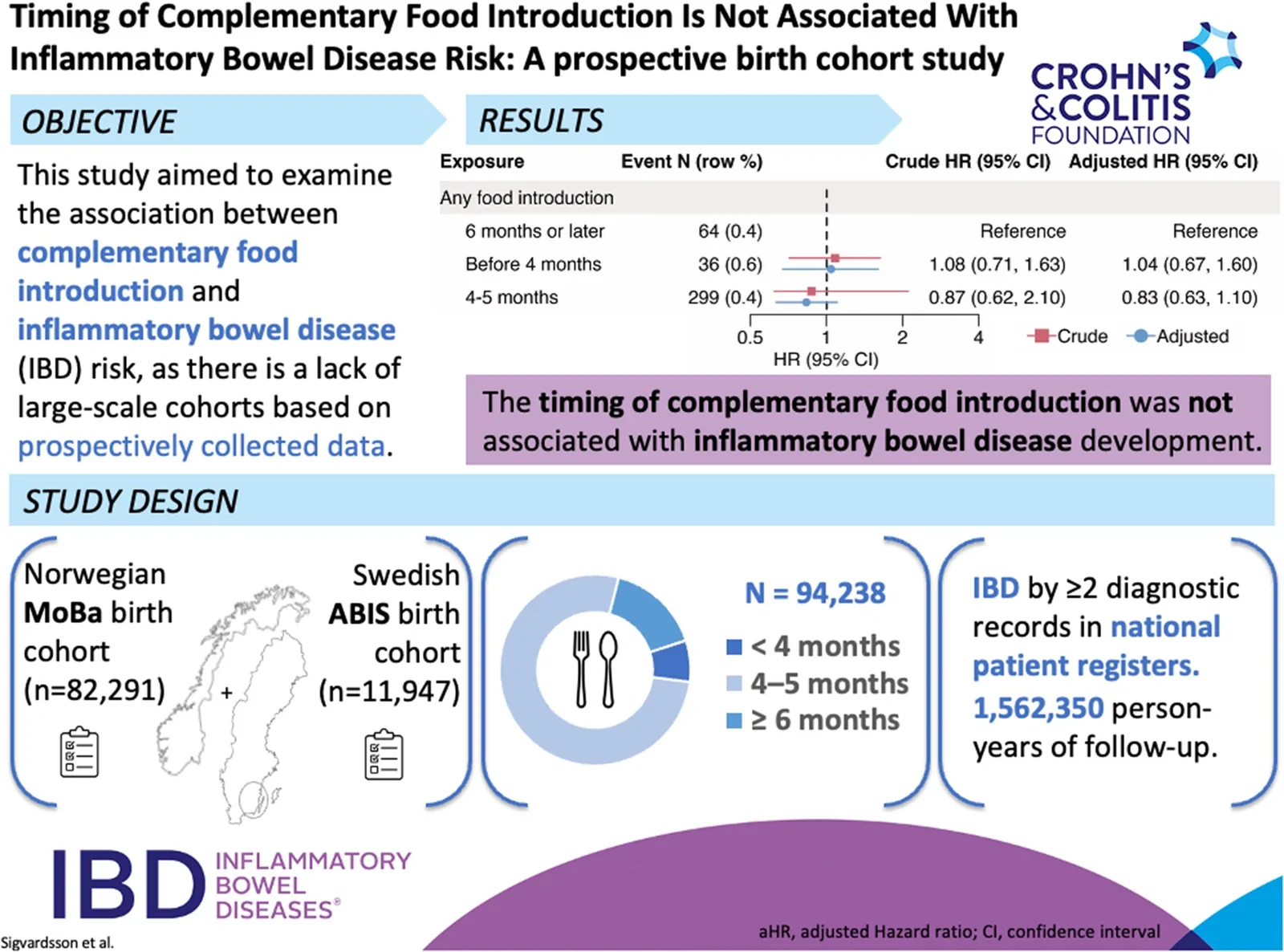

Key messages **What is already known?** 

• Complementary food introduction shapes gut immune development, and its alterations may affect inflammatory bowel disease (IBD) risk; however, prospective data for robust risk estimation are lacking.

**What is new here?** 

• In this first binational birth cohort study using prospectively collected data, neither overall timing nor patterns of complementary food introduction across major food groups were associated with IBD.

**How can this study help patient care?** 

• Our consistent null findings provide reassuring evidence that the timing of complementary food introduction does not affect IBD risk; other environmental factors might be more important in IBD etiology.

## Introduction

Inflammatory bowel diseases (IBDs), including Crohn’s disease (CD) and ulcerative colitis (UC), are chronic gastrointestinal diseases likely caused by an interplay between genetic susceptibility, immunological, epigenetic, and environmental factors.^1^ The rising incidence and compounding prevalence of IBD globally underscores the need to identify modifiable risk factors to mitigate the increasing burden of the disease.^2^^,^^3^ The role of early-life diet has been suggested as one of the top priorities in future IBD research,^4^ and for potential IBD prevention.^5^

The age and pattern of food introduction at weaning are vital for the developing gut intestinal flora and the gut immune tolerance,^6^ which are factors believed to influence IBD development.^5^ Still, few studies have examined the timing of complementary feeding (ie, food introduction besides formula and breast milk),^7^ on later IBD risk (Table S1, describing earlier studies). Existing evidence is limited to small-scale, retrospectively designed studies that are underpowered and liable to recall bias, limiting the ability to exclude all but the most prominent risk factors. Further, no previous data have examined food introduction trajectories on IBD risk.

The current Nordic nutritional guidelines recommend complementary food introduction at 6 months of age and gradually increasing the food consistency and diversity as the infant ages. Complementary food introduction before 4 months of age is not recommended.^8^ However, the more precise timing and sequence of introduction across food groups (ie, cereals, dairy, etc.) vary considerably. The global recommendations (by the World Health Organization) suggest introducing complementary foods from age 6 months^7^, although that has been debated.^9^ Both Nordic and global recommendations encourage continued breastfeeding or formula feeding during the complementary introduction period.

Recognizing the knowledge gap of prior IBD research, this binational prospective birth cohort study aimed to assess the association between the timing and pattern of complementary food introduction and the risk of later IBD.

## Methods

### Study population

We used data from 2 birth cohorts: All Babies In Southeast Sweden (ABIS) and the Norwegian Mother, Father, and Child Cohort Study (MoBa).^10^ The cohorts followed children from birth until young adulthood to examine risk factors for disease development by collecting questionnaire data and individual-level linkages to national health registers. The ABIS and MoBa cohorts were approved by the research ethics committees of Sweden and Norway, respectively. The parents gave written informed consent for their children when entering the cohorts.

Out of 21 700 children born in southeast Sweden (Småland, Blekinge, Östergötland, and Öland) from October 1, 1997, until October 1, 1999, 17 055 children were included in ABIS (participation rate 79%). MoBa, conducted by the Norwegian Institute of Public Health, included 114 000 children born across Norway between 1999 and 2009 (participation rate 41%). This study included 94 238 participants from ABIS (n = 11 947) and MoBa (n = 82 291) (Figure 1) with a valid personal identity number and data on complementary feeding, as described below.

**Figure 1:**
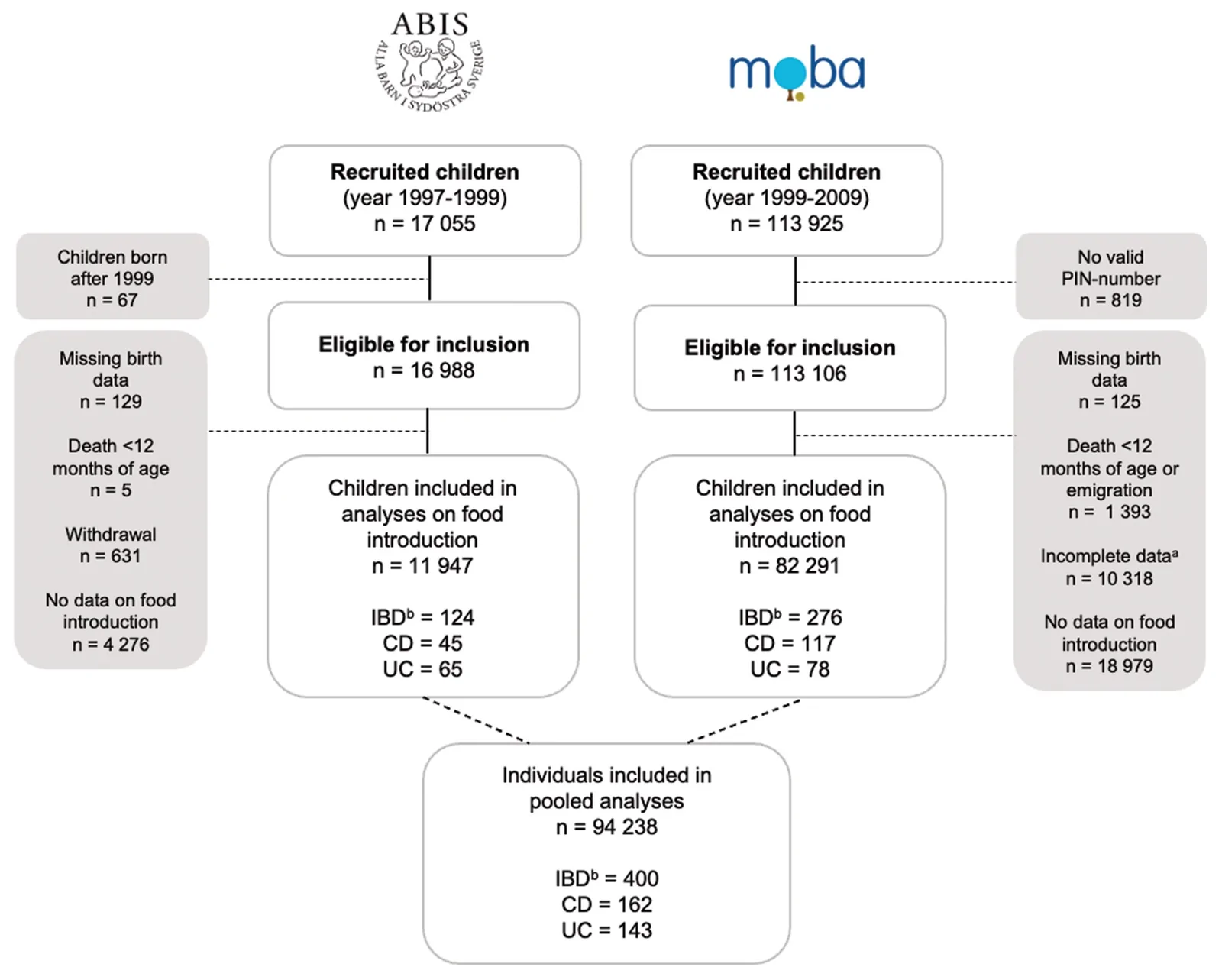
Flowchart of the study populations of the All Babies in Southeast Sweden (ABIS) and Norwegian Mother, Father and Child Cohort Study (MoBa) cohorts. Participants were followed from birth through national health registers. ^a^Gave consent but did not complete questionnaires and were excluded. ^b^Includes inflammatory bowel disease (IBD) unclassified events. CD, Crohn’s disease; PIN, personal identification number; UC, ulcerative colitis.

## Ethical approval

The parents gave written informed consent for their children when entering the cohorts. In MoBa, the participants gave their consent to participate in follow-up when turning 18 years of age. In ABIS, the participants consented to follow-ups around 17 to 19 years of age and in adulthood in connection to responding to questionnaires. ABIS was approved by the Research Ethics Committees of the Faculty of Health Sciences, Linköping University (Li 287-96) and the Medical Faculty of Lund University (Lu 83-97), with ethical approvals (Dnr: 96287; 03-092; 03-513; 2018/380-32; 1849/2021). The establishment of MoBa and initial data collection were based on a license from the Norwegian Data Protection Agency and approval from the Regional Committees for Medical and Health Research Ethics (no. 153328). All data were stored safely with two-factor authentication.

### Exposures: Timing and pattern of complementary food introduction

We examined the timing of any complementary food^7^ defined by the earliest reported age, and the risk of later IBD in the ABIS and MoBa cohorts, as well as the IBD risk related to the introduction of individual food groups and various patterns of introduction across food groups.

#### Timing of any complementary food introduction

In ABIS, a diary administered in the postnatal ward was completed prospectively during the first 12 months of life, covering the introduction (date) of 25 food items (Table S2, describing exposure data). The overall timing of complementary food introduction (ie, the first age of any food introduced besides formula and breast milk) was primarily retrieved from the 0- to 12-month food diary and secondarily from a questionnaire administered at the child’s age of 12 months. In MoBa, a questionnaire distributed around 6 months of age covered the age (months) when 16 food items were given to the child for the first time (Table S2). Data were additionally retrieved from a questionnaire at 18 months for dairy introduction in MoBa. To rule out the influence on results from erroneously recorded data from the 6-month questionnaire in MoBa, this study did not consider overall complementary food introduction before 3 months or later than 9 months of age. Questionnaires were administered and completed primarily by the mothers at different time points, depending on the cohort study protocols and the child’s age (Table S2). An overview of the data collection is presented in Figure S1, which shows the time points of data collection in ABIS and MoBa.

#### Individual food groups and patterns of complementary food introduction

We clustered infant food items into 7 food groups: any cereals (with or without gluten), cereals with gluten, fruits and berries, vegetables and potatoes, meat, fish, and dairy. These food groups were used to identify patterns of complementary food introduction, described subsequently and in Supplemental Material 1. The age at which each food group was introduced was defined by the earliest reported age of any included food item. Based on data distribution and current complementary feeding guidelines,^7^^,^^9^ complementary food introductions were categorized as <4 months, 4 to 5 months, and ≥6 months.

### Outcomes

Cohort data were linked to the Swedish and Norwegian National Patient Registers^11^^,^^12^ using the personal identification number assigned to all Swedish and Norwegian residents. These registries contain diagnostic data from inpatient and specialized outpatient care. The outcomes of IBD included events of CD, UC, and IBD unclassified. We defined IBD as at least 2 International Classification of Diseases–Tenth Revision codes for the disease recorded by the end of 2021 (MoBa) and 2023 (ABIS) (Table S3, describing the outcome definitions). This definition has, on medical record reviews, been shown to have a positive predictive value of ≥93% for a clinical diagnosis of IBD in Sweden^13^ and Norway.^14^ The time of an IBD diagnosis equaled the time of the first International Classification of Diseases code for IBD, with high accuracy in the Swedish National Patient Register.^15^

### Other data

We accounted for factors that may influence the timing of complementary food introduction and IBD risk. Covariates were retrieved from questionnaires and the Medical Birth Registry of Sweden and Norway.^16^^,^^17^ This included data on the child’s sex, child’s birth year, child’s birth weight, delivery mode, child’s gestational age, child’s diet diversity at age 1 year,^18^ child’s antibiotic use by 1 year age,^19^ maternal smoking during pregnancy,^20^^,^^21^ maternal age^21^ and education level^22^ at birth, any breastfeeding duration,^21^ the start of formula feeding,^23^ parental IBD, and parental origin (Figure S2, showing a directed acyclic graph). Covariates were categorized as shown in Table 1 and are further described in Supplemental Material 2.

**Table 1:** Participant characteristics in the ABIS and MoBa birth cohorts according to the timing of any complementary food introduction.

	ABIS				MoBa			
	All (N = 11 947)	<4 mo (n = 1341)	4-5 mo (n = 9453)	≥6 mo (n = 1153)	All (N = 82 291)	<4 mo (n = 4858)	4-5 mo (n = 62 453)	≥6 mo (n = 13 637)
**Sex**								
** Female**	5738 (48.0)	612 (45.6)	4607 (48.7)	519 (45.0)	40 163 (48.8)	2111 (43.5)	30 250 (48.4)	7120 (52.2)
** Male**	6209 (52.0)	729 (54.4)	4846 (51.3)	634 (55.0)	42 128 (51.2)	2747 (56.5)	32 203 (51.6)	6517 (47.8)
**Age at the end of follow-up, y^a^**								
** Mean ± SD**	25.2 ± 1.1	25.2 ± 1.4	25.2 ± 1.1	25.3 ± 1.1	16.5 ± 2.2	17.0 ± 2.2	16.4 ± 2.2	16.4 ± 2.1
** Median (IQR)**	25.3 (24.8-25.8)	25.2 (24.8-25.7)	25.3 (24.8-25.7)	25.5 (25.0-25.8)	16.2 (14.8-18.2)	16.9 (15.2-18.7)	16.2 (14.7-18.1)	16.2 (14.7-18.0)
**Follow-up time, y^b^**								
** Mean ± SD**	24.2 ± 1.1	24.2 ± 1.4	24.2 ± 1.1	24.3 ± 1.1	15.5 ± 2.2	16.0 ± 2.2	15.4 ± 2.2	15.4 ± 2.1
** Median (IQR)**	24.3 (23.8-24.8)	24.2 (23.7-24.8)	24.3 (23.7-24.8)	24.5 (24.0-24.8)	15.2 (13.7-17.2)	15.9 (14.2-17.7)	15.2 (13.7-17.1)	15.2 (13.7-17.0)
**Calendar year of birth**								
** 1997**	1329 (11.1)	134 (10.0)	1041 (11.0)	154 (13.4)	—	—	—	—
** 1998**	6311 (52.8)	665 (49.6)	4960 (52.5)	686 (59.5)	—	—	—	—
** 1999**	4307 (36.1)	542 (40.4)	3452 (36.5)	313 (27.1)	23 (<0.1)	3 (0.1)	12 (<0.1)	2 (<0.1)
** 2000**	—	—	—	—	1604 (1.9)	151 (3.1)	1238 (2.0)	183 (1.3)
** 2001**	—	—	—	—	3213 (3.9)	258 (5.3)	2406 (3.9)	483 (3.5)
** 2002**	—	—	—	—	6791 (8.3)	541 (11.1)	5016 (8.0)	1074 (7.9)
** 2003**	—	—	—	—	9963 (12.1)	739 (15.2)	7425 (11.9)	1617 (11.9)
** 2004**	—	—	—	—	10 609 (12.9)	678 (14.0)	7964 (12.8)	1792 (13.1)
** 2005**	—	—	—	—	12 039 (14.7)	653 (13.4)	9073 (14.5)	2107 (15.5)
** 2006**	—	—	—	—	13 350 (16.2)	733 (15.1)	10 199 (16.3)	2228 (16.3)
** 2007**	—	—	—	—	12 093 (14.7)	607 (12.5)	9295 (14.9)	2026 (14.9)
** 2008**	—	—	—	—	10 084 (12.3)	421 (8.7)	7820 (12.5)	1712 (12.6)
** 2009**	—	—	—	—	2522 (3.1)	74 (1.5)	2005 (3.2)	413 (3.0)
**IBD diagnosis^c^**								
** IBD**	124 (1.0)	13 (1.0)	99 (1.0)	12 (1.0)	276 (0.3)	23 (0.5)	200 (0.3)	52 (0.4)
** CD**	45 (0.4)	4 (0.3)	36 (0.4)	5 (0.4)	117 (0.1)	12 (0.2)	86 (0.1)	19 (0.1)
** UC**	65 (0.5)	7 (0.5)	53 (0.6)	5 (0.4)	78 (0.1)	3 (0.1)	60 (0.1)	15 (0.1)
**Food introduction timing by LCA pattern**	n = 9384	n = 983	n = 7526	n = 1153	n = 82 246	n = 4856	n = 62 432	n = 13 615
** Early introducers**	375 (3.1)	375 (28.0)	0 (0.0)	0 (0.0)	2379 (2.9)	2379 (49.0)	0 (0.0)	0 (0.0)
** Mid introducers**	4046 (33.9)	510 (38.0)	3536 (37.2)	0 (0.0)	18 927 (23.0)	1410 (29.0)	17248 (27.6)	1 (<0.1)
** Mid-late introducers**	2611 (21.9)	42 (3.1)	2569 (27.2)	0 (0.0)	44 004 (53.5)	562 (11.6)	43 438 (69.6)	0 (0.0)
** Late introducers**	2352 (19.7)	56 (4.2)	1421 (15.0)	875 (75.9)	16 936 (20.6)	505 (10.4)	1746 (2.8)	13 614 (99.8)
**Start of formula feeding (%)**	n = 4817	n = 723	n = 3822	n = 272	n = 54 194	n = 4278	n = 43 635	n = 5650
** <1 mo**	1540 (32.0)	275 (38.0)	1179 (30.8)	86 (31.6)	17 572 (32.4)	1548 (36.2)	13 977 (32.0)	1833 (32.4)
** 1-3 mo**	1893 (39.3)	353 (48.8)	1444 (37.8)	96 (35.3)	12 345 (22.8)	1580 (36.9)	9967 (22.8)	671 (11.9)
** ≥4 mo**	1384 (28.7)	95 (13.1)	1199 (31.4)	90 (33.1)	24 277 (44.8)	1150 (26.9)	19 691 (45.1)	3146 (55.7)
**Any breastfeeding duration^d^**	n = 10 978	n = 1216	n = 8743	n = 1019	n = 82 291	n = 4858	n = 62 453	n = 13 637
** 0 to <4 mo**	1468 (13.4)	437 (35.9)	976 (11.2)	55 (5.4)	10 638 (12.9)	2077 (42.8)	8117 (13.0)	308 (2.3)
** 4 to <6 mo**	1113 (10.1)	209 (17.2)	865 (9.9)	39 (3.8)	5903 (7.2)	619 (12.7)	4871 (7.8)	274 (2.0)
** ≥6 mo**	8397 (76.5)	570 (46.9)	6902 (78.9)	925 (90.8)	65 750 (79.9)	2162 (44.5)	49 465 (79.2)	13 055 (95.7)
**Maternal age at delivery^e^**	n = 11 752	n = 1319	n = 9295	n = 1138	n = 82 248	n = 4858	n = 62 424	n = 13 624
** <25 y**	1689 (14.4)	295 (22.4)	1301 (14.0)	93 (8.2)	8326 (10.1)	1217 (25.1)	6276 (10.1)	700 (5.1)
** 25-34 y**	8586 (73.1)	860 (65.2)	6897 (72.2)	829 (72.8)	59 624 (72.5)	3122 (64.3)	45 853 (73.5)	9766 (71.7)
** ≥35 y**	1477 (12.6)	164 (12.4)	1097 (11.8)	216 (19.0)	14 298 (17.4)	519 (10.7)	10 295 (16.5)	3158 (23.2)
**Parental IBD**	n = 11 947	n = 1341	n = 9453	n = 1153	n = 82 291	n = 4858	n = 62 453	n = 13 637
** No**	11 795 (98.7)	1321 (98.5)	9335 (98.8)	1139 (98.8)	80 391 (97.7)	4741 (97.6)	60 998 (97.7)	13 353 (97.9)
** Yes**	152 (1.3)	20 (1.5)	118 (1.2)	14 (1.2)	1900 (2.3)	117 (2.4)	1455 (2.3)	284 (2.1)
**Parental origin^f^**	n = 11 691	n = 1291	n = 9283	n = 1117	n = 78 848	n = 4592	n = 59 779	n = 13 198
** Norwegian/Swedish**	10 568 (90.4)	1109 (85.9)	8480 (91.3)	979 (87.6)	71 077 (90.1)	4239 (92.3)	53 930 (90.2)	11 774 (89.2)
** Other**	1123 (9.6)	182 (14.1)	803 (8.7)	138 (12.4)	7771 (9.9)	353 (7.7)	5849 (9.8)	1424 (10.8)
**Maternal education level^g^**	n = 11 680	n = 1291	n = 9277	n = 1112	n = 81 974	n = 4835	n = 62 211	n = 13 591
** Low**	840 (7.2)	196 (15.2)	563 (6.1)	81 (7.3)	5577 (6.8)	918 (19.0)	3999 (6.4)	546 (4.0)
** Medium**	6496 (55.6)	761 (58.9)	5152 (55.5)	583 (52.4)	23 576 (28.8)	2218 (45.9)	18 132 (29.1)	2868 (21.1)
** High**	4344 (37.2)	334 (25.9)	3562 (38.4)	448 (40.3)	52 821 (64.4)	1699 (35.1)	40 080 (64.4)	10 177 (74.9)

**Values are n (%), unless otherwise indicated.:** See Table S4 for details on missing data.

Abbreviations: ABIS, All Babies in Southeast Sweden; CD, Crohn’s disease; IBD, inflammatory bowel disease; IQR, interquartile range; LCA, latent class analysis; MoBa, Norwegian Mother, Father, and Child Cohort Study; UC, ulcerative colitis.

a Age at the end of the study equals age at diagnosis for IBD, CD, and UC events.

b Follow-up started at 1 year.

c Includes IBD unclassified.

d Partial or exclusive breastfeeding duration.

e Categories were merged for the table presentation, and the following categories were used in the analyses: 15 to 19/20 to 24/25 to 29/30 to 34/35 to 39/40 to 44 years.

f For ABIS, other country indicates if any parent was born in another country than Sweden; in MoBa, it indicates any parent having a different native language than Norwegian.

g High education level refers to ≥13 years of education in both cohorts, medium education level refers to 12 years of education in both cohorts, and low education level refers to 9 years in ABIS and 9-11 years in MoBa.

### Statistical analyses

The association between complementary food introduction and IBD risk was evaluated using Cox proportional hazards regression, adjusting for the child’s sex and birth year, maternal age and education at birth, parental history of IBD, and parental origin. The start of follow-up was set at 12 months of age; events occurring before this age were excluded. Cluster-robust standard errors were used to account for dependencies between siblings. Cohort-specific hazard ratios were pooled using the DerSimonian-Laird random-effects meta-analysis method.^24^ Heterogeneity between cohorts was evaluated using the Cochran Q test, which showed low heterogeneity for all IBD analyses (*P* > .10).^25^

To identify patterns in the timing of food group introduction, latent class analysis (LCA)^26^ was performed using ABIS data, in which food group data completeness was higher; these patterns (latent classes) were subsequently applied to the MoBa data. The number of latent classes was determined based on the Bayesian information criterion. A 4-class solution, representing early, mid, mid-late, and late introduction across most food groups, was selected for its interpretability and statistical power (Supplemental Material 1, providing a full description of the LCA results).

Two sensitivity analyses examined the consistency of our findings after additionally adjusting for (1) any breastfeeding duration and the start of formula feeding and (2) maternal smoking during pregnancy,^20^ child’s birth weight, delivery mode, child’s gestational age, child’s diet diversity at 1 year of age,^18^ and the child’s antibiotic use by 1 year of age.^19^ We also examined IBD risk by stratifying the population by breastfeeding duration (<6 months/≥6 months). Finally, secondary analyses were restricted to the outcome of childhood-onset IBD (<18 years of age).

Statistical analyses were performed using IBM SPSS Statistics version 29 and R version 4.4.2 (R Foundation for Statistical Computing), with the R packages poLCA, metafor, and meta.

## Results

### Study population

Over a total follow-up of 1 562 350 person-years (289 312 in ABIS and 1 273 038 in MoBa), 400 individuals developed IBD (ABIS, n = 124; MoBa, n = 276). The mean age at the end of follow-up was 25.2 years in ABIS and 16.5 years in MoBa (Table 1). Baseline characteristics were mainly similar in the ABIS and MoBa populations (Table 1 and Table S4, the latter describing participant characteristics by the presence of IBD). The incidence rate (IR) for IBD per 100 000 person-years was 21.7 in MoBa; standardizing IRs in ABIS to align with the age and sex distribution in MoBa resulted in an IR of 23.4 in ABIS (Table S5, showing follow-up times and incidence rates). Generally, those with missing data had mothers with a lower education level and were more likely to smoke during pregnancy (Table S6, showing participant characteristics of those with valid and missing data). In ABIS, the IR for IBD was slightly lower for those missing complementary food data, while no difference was found in MoBa.

### Pooled estimates

#### Any complementary food introduction

We included 92 894 participants in the pooled analyses of any complementary food introduction (ABIS, n = 11 947; MoBa, n = 80 947). Most were introduced to complementary food at 4 to 5 months of age (n = 71 905 [77%]), typically starting with vegetables/potatoes (ABIS) or cereals (MoBa). The timing of any complementary food introduction yielded an adjusted hazard ratio (aHR) of 1.04 (95% confidence interval [CI], 0.67-1.60) for introduction <4 months of age and aHR of 0.83 (95% CI, 0.63-1.10) for introduction at 4 to 5 months of age, compared with ≥6 months age (Figure 2), after accounting for child’s sex and birth year, maternal age and education level at birth, parental IBD, and parental origin. Sensitivity analyses additionally accounting for any breastfeeding duration and the start of formula feeding showed essentially similar estimates (<4 months: aHR, 1.16 [95% CI, 0.64-2.08]; 4-5 months: aHR, 0.82 [95% CI, 0.52-1.27]; compared with ≥6 months), as did sensitivity analyses adjusted for perinatal factors (Table S7, presenting pooled estimates for sensitivity analyses). There were no statistically significant associations between the introduction of any complementary food and IBD among those who were breastfed for at least 6 months (Table S8, presenting estimates by breastfeeding duration ≥6 months). Analyses among children breastfed for <6 months were not possible due to no IBD events among those introduced to complementary feeding ≥6 months in MoBa. Pooled analyses also revealed no associations between any complementary food introduction and the development of CD and UC (Figures 3 and 4) or for the outcome of childhood-onset IBD (<18 years) (Table S9, presenting pooled estimates for childhood-onset IBD).

**Figure 2:**
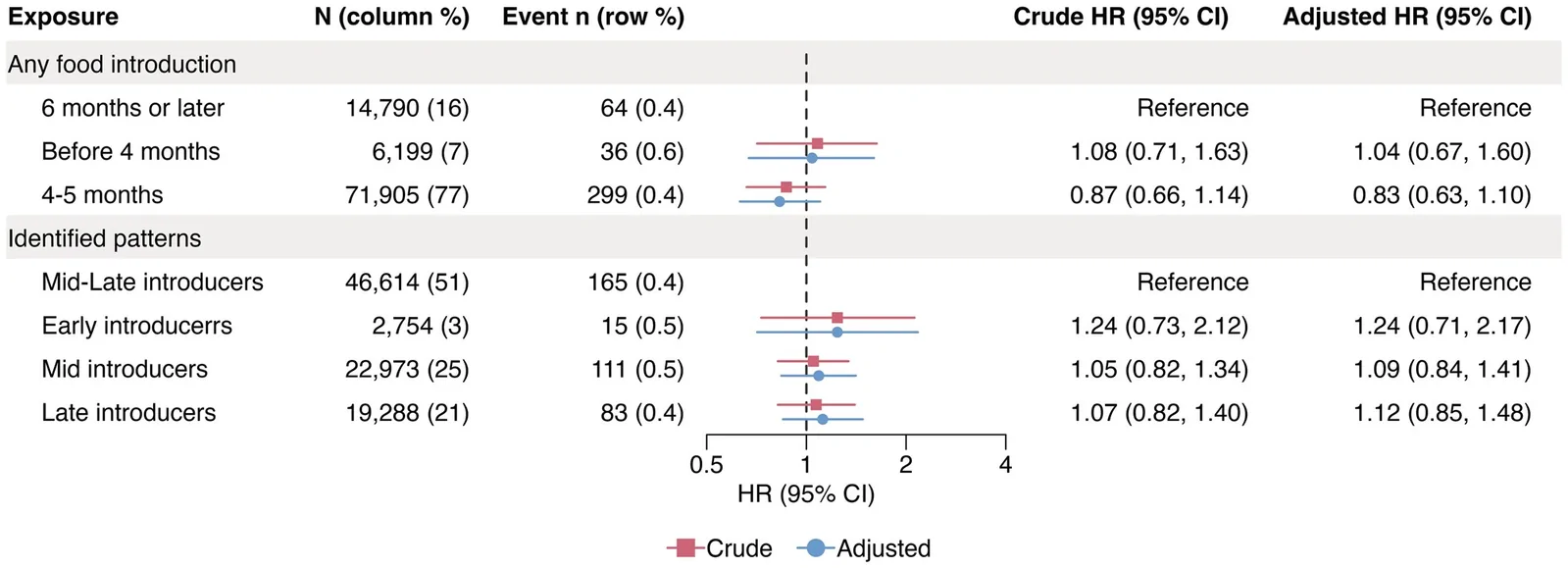
Pooled hazard ratios (HRs) for inflammatory bowel disease by the timing of any complementary food introduction and patterns of food group introduction. Points represent HRs; horizontal lines show 95% confidence intervals (CIs). Estimates are presented for both unadjusted (crude) and adjusted models. The adjusted model included the child’s sex and birth year, maternal age and education level, parental inflammatory bowel disease, and parental origin.

**Figure 3:**
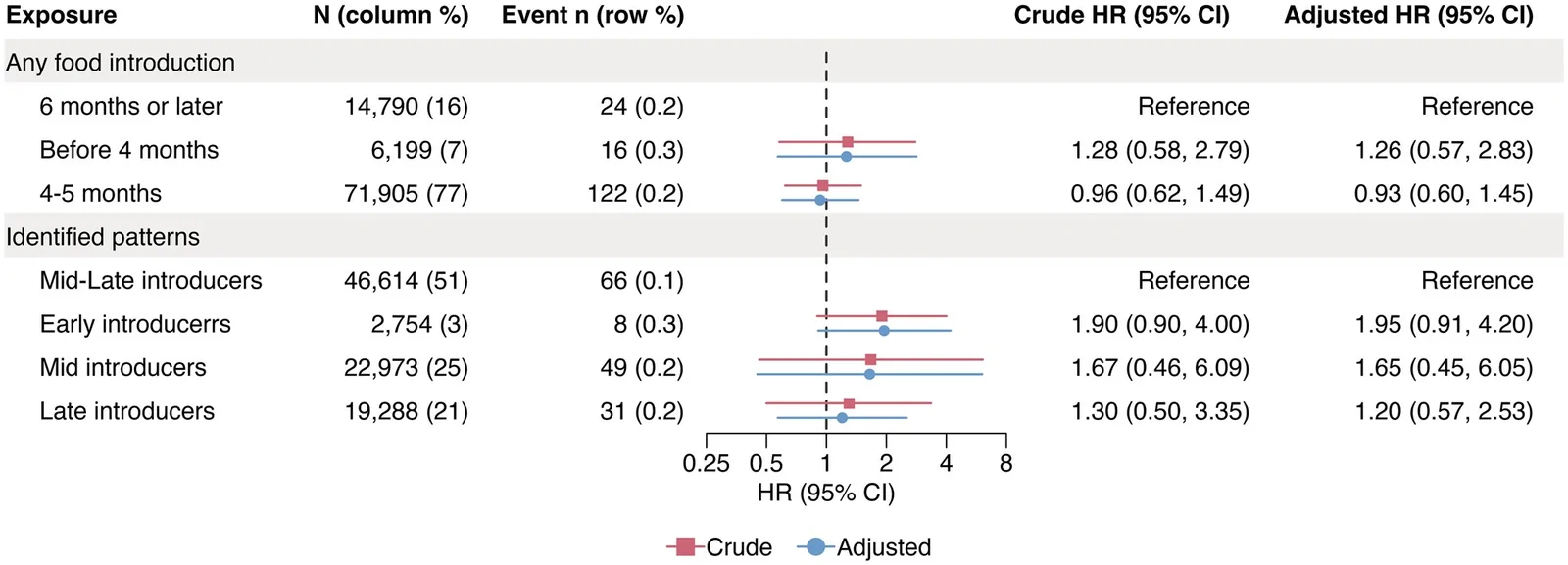
Pooled hazard ratios (HRs) for Crohn’s disease by the timing of any complementary food introduction and patterns of food group introduction. Points represent HRs; horizontal lines show 95% confidence intervals (CIs). Estimates are presented for both unadjusted (crude) and adjusted models. The adjusted model included the child’s sex and birth year, maternal age and education level, parental inflammatory bowel disease, and parental origin.

**Figure 4:**
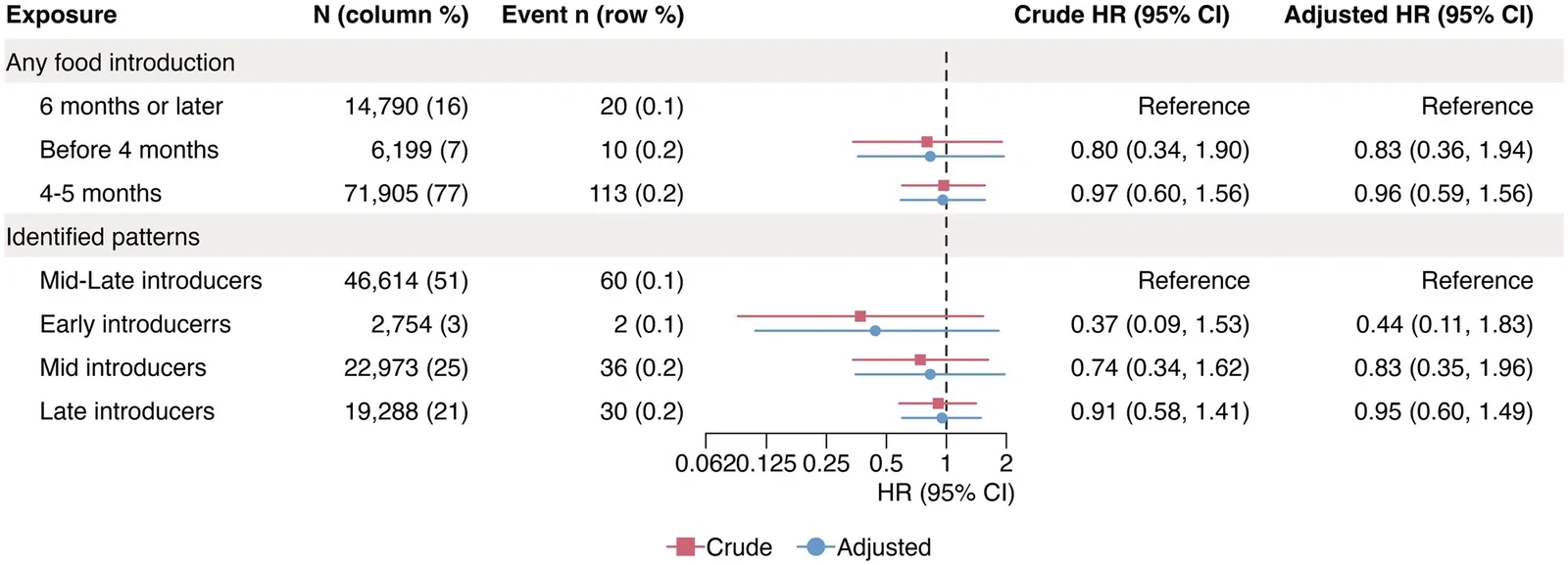
Pooled hazard ratios (HRs) for ulcerative colitis by the timing of any complementary food introduction and patterns of food group introduction. Points represent HRs; horizontal lines show 95% confidence intervals (CIs). Estimates are presented for both unadjusted (crude) and adjusted models. The adjusted model included the child’s sex and birth year, maternal age and education level, parental inflammatory bowel disease, and parental origin.

#### Patterns of complementary food introduction identified by LCA

A total of 91 630 participants with food group introduction data were included in the LCA, identifying 4 patterns of complementary feeding: early, mid, mid-late, and late introduction. Most children were classified as mid-late introducers (n = 46 614 [51%]), characterized by the introduction of most food groups at 4 to 5 months or ≥6 months. The risk of IBD did not differ across the identified patterns. Compared with the mid-late group: aHR, for IBD were 1.24 (95% CI, 0.71-2.17) for early, 1.09 (95% CI, 0.84-1.41) for mid, and 1.12 (95% CI, 0.85-1.48) for late introduction pattern (Figure 2). Pooled analyses for CD and UC yielded similar null associations (Figures 3 and 4).

#### Individual food groups

Across all food groups, fish tended to be introduced later than other foods, with a mean age of 7.6 months in ABIS and 5.8 months in MoBa (Table S10, describing frequencies and mean ages for food group introductions). The risk of IBD did not differ by the timing of individual food groups (Table S11, presenting pooled estimates for individual food groups and IBD risk).

### Cohort-specific analyses

Cohort-specific analyses were largely consistent with the pooled results (Tables S12, S13, and S14, presenting cohort-specific estimates for IBD, CD, and UC, respectively). For IBD, complementary food introduction <4 months vs ≥6 months of age yielded an aHR of 0.91 (95% CI, 0.41-2.01) in ABIS and 1.09 (95% CI, 0.65-1.84) in MoBa (Table S12). The estimates for IBD and UC (Table S14) by identified patterns of complementary food introduction were consistent and nonsignificant across both cohorts. Also, CD estimates were not significant in MoBa. However, in ABIS 22 of 4046 mid introducers developed CD (aHR, 3.54 [95% CI, 1.20-10.41] vs mid-late). This association did not remain statistically significant when accounting for breastfeeding duration and the start of formula feeding (Table S13).

## Discussion

This is likely the first study using prospectively collected data from infant questionnaires and food diaries to examine the timing (age) and patterns of complementary food introduction with the risk of later IBD. In this binational birth cohort, neither the timing of any complementary feeding, the introduction of individual food groups nor the identified patterns of food group introduction were associated with IBD risk. Similarly, null findings were observed in sensitivity analyses across cohorts and for the outcomes of CD and UC subtypes, overall indicating that the timing of complementary food introduction is no major risk factor for IBD development.

The start of complementary feeding around 6 months of age is likely beneficial for short-term health consequences such as early growth and infection susceptibility.^27^ Still, the evidence is insufficient to recommend an optimal age and timing across major food groups for long-term health and disease prevention overall. The first thousand days of life shape intestinal microbiota development, highly influenced by early feeding practices.^28^ Already at 3 years of age, the gut microbiome stabilizes in a more adult-like pattern.^5^ Even though the microbiome has been widely discussed concerning IBD,^29^ there is insufficient evidence to establish whether dysbiosis in the microbiome causes or is the consequence of intestinal inflammation. The introduction of complementary food, which can profoundly influence the gut microbiome composition and diversity,^30^ has been proposed as a candidate risk factor for IBD.^5^

Our results align with the few earlier publications in this field (Table S1). Already in 1989^31^ and 1991,^32^ Koletzko et al found no significant differences in age at solid food introduction between patients with CD or UC and control subjects, based on retrospective questionnaire data. Similarly, Gilat et al^33^ reported no association between the timing of mixed feedings in infancy and IBD risk in a multinational study of 499 patients with IBD with disease onset <20 years of age. In a population-based case-control study, Baron et al^34^ found no differences in the age of flour, meat, or vegetable introduction between individuals with IBD, diagnosed between 1988 and 1997, and control subjects. More recently, Fantodji et al^35^ observed no significant association between solid food introduction before 6 months of age and CD or UC risk in a Canadian nested case-control study of over 1700 events of IBD. Overall, systematic reviews and meta-analyses have not found complementary feeding associated with IBD,^36^^,^^37^ and the few previous studies are restricted to retrospectively collected data.^31–35^ For example, while Fantodij et al included over 1700 events of IBD, information on solid food introduction was collected around 50 years after its occurrence. This study used data reported from birth until 18 months of age, thus in close connection to the start of complementary feeding. Therefore, our study contributes by showing that previous null findings do not arise from erroneously reported data, causing imprecise exposure assessment. Additionally, the definition of food introduction varies in previous studies. We defined food introduction according to World Health Organization,^7^ while others have examined related exposures, such as the introduction of allergenic foods specifically, the introduction of solid foods only, or solely exclusive breastfeeding duration. The introduction of gluten or allergenic foods, rather than overall complementary food introduction, has been widely studied as a risk factor for other immune-mediated diseases^38^ such as celiac disease, conditions that partly share etiological and epidemiological traits with IBD.^39^

In this study, the timing of complementary foods reflects the first time (age) that the child received another nutritional source than breastfeeding or formula feeding. On the other hand, the patterns of food group introduction reflect food introduction trajectory across major infant food groups. Theoretically, these 2 exposures may overlap for infants introduced to most food groups at the same age.

### Strengths and limitations

This study has some major strengths. The nature of prospective data limits the risk of reverse causation and recall bias, which may hamper causal inference of data retrospectively collected at the time of IBD diagnosis. Our binational cohort design allowed us to examine complementary food introduction in >90 000 children and link data to high-quality national patient registers.^11^^,^^12^ Our detailed exposure assessment permitted the first investigation of patterns of food introduction in relation to later IBD risk. Additionally, our validated IBD definition has shown a positive predictive value of ≥93% in both cohorts.^13^^,^^14^ Another strength compared with previous studies in this field is our ability to account for several potential confounders, such as parental IBD, breastfeeding duration, formula feeding, and sociodemographic factors,^22^ as well as maternal smoking in pregnancy,^20^ early-life antibiotic use,^19^ and the child’s dietary diversity at 1 year of age.^18^

Despite 2 large cohorts, the number of IBD events and missing data for individual food groups limits the power of our analyses, which prevents us from confidently ruling out more modest associations between the timing of complementary food introduction and later IBD risk. Additionally, given the follow-up time of our cohorts (mean age at end of study was 25.2 years in ABIS and 16.5 years in MoBa), we could only identify IBD outcomes developing in childhood and young adulthood. Hence, we do not know whether complementary feeding may contribute to IBD development later in life. The relatively young age of participants has also reduced the total number of IBD events, thereby limiting our statistical power. This study does not exclude the possibility of stronger associations related to more extreme feeding patterns, such as very early or very late food introduction, or more detailed classifications of food introduction patterns (eg, only cereals introduced early). LCA accommodates some missingness through model-based estimation of class membership and assumes data are missing at random, which may not fully capture systematic reporting differences. We cannot rule out that erroneous parental recall of the timing of food introduction may have contributed to misclassification of the timing of complementary food introduction. Importantly, such misclassification is unlikely to be related to future IBD risk and therefore unlikely to cause spurious associations, although it may have attenuated the estimates toward the null.

The recommendations on exclusive breastfeeding duration and complementary food introduction in Sweden and Norway are largely similar and based on local and global recommendations, which have been updated and, to some extent, changed over the study time.^7^^,^^8^ While breastfeeding (or formula feeding) is advised as the best nutritional source for the first 6 months of life, Swedish and Norwegian guidelines support introduction of small tastes of complementary foods from 4 to 6 months of age, depending on the child’s nutritional need, perceived interest, and readiness.^8^ Updates to official recommendations between 1997 and 2009 could have influenced feeding practices, which we accounted for by adjusting for birth year. In our study, we found that those introduced to complementary foods earlier (<4 months) than the generally recommended ages had a greater proportion of mothers with low educational levels and who were young (<25 years), compared with those introduced to complementary foods later. While our estimates were adjusted for maternal age and education level, children with different timing of complementary food introduction may differ with respect to other unmeasured lifestyle or dietary factors that could influence IBD risk, such as high consumption of processed foods, which have been suggested as a candidate risk factor for IBD.

The mothers in MoBa are older and less likely to smoke than mothers in the general population.^10^ It is unknown how our results reflect underrepresented groups. Due to differences in geographical, socioeconomic, and dietary factors,^40^ our results originating from 2 Scandinavian countries might not be generalizable to countries with other complementary food introduction practices and IBD risks.

## Conclusion

To our knowledge, this is the first large-scale study using prospective data to examine the association between complementary food introduction and the risk of IBD. Our findings do not support that timing or patterns of complementary food introduction are major risk factors for IBD development.

## Supplementary Material

izag093_Supplementary_Data

## Data Availability

No additional data are available due to Swedish and Norwegian legal and data protection regulations. Information about research and data access from the Norwegian Mother and Child Cohort Study can be found here: https://www.fhi.no/en/ch/studies/moba/for-forskere-artikler/research-and-data-access/.
