# Supplementary material for: Timing of complementary food introduction is not associated with inflammatory bowel disease risk: A prospective birth cohort study
Source: Inflamm Bowel Dis. 2026 Jun 9;32(10):1937–48. doi: 10.1093/ibd/izag093 (PMC13626236; doi:10.1093/ibd/izag093)
Supplement: Supplementary file 1 [file izag093_supplementary_data.docx]

Supplementary Information.

*Timing of Complementary Food Introduction Is Not Associated With Inflammatory Bowel Disease Risk: A prospective birth cohort study.*

[Supplementary Tables.	2](#_Toc224893971)

[Supplementary Table 1. Earlier publications on complementary or solid food introduction and risk of inflammatory bowel disease.	2](#_Toc224893972)

[Supplementary Table 2. Exposure definitions of measures of food introduction in ABIS and MoBa cohorts, and the harmonization of these variables.	4](#_Toc224893973)

[Supplementary Table 3. Register-based definition of inflammatory bowel disease.	10](#_Toc224893974)

[Supplementary Table 4. Characteristics of participants by IBD, CD & UC.	11](#_Toc224893975)

[Supplementary Table 5. Person-years of follow-up, crude incidence rates, and age-and-sex standardized incidence rates for inflammatory bowel disease in ABIS and MoBa cohorts.	13](#_Toc224893976)

[Supplementary Table 6. Characteristics of those with valid data and missing data on complementary food introduction in the ABIS and MoBa cohorts.	14](#_Toc224893977)

[Supplementary Table 7. Sensitivity analyses: pooled estimates for the timing of complementary food introduction and inflammatory bowel disease risk, including adjustment for breastfeeding duration and start of formula feeding, or perinatal factors.	15](#_Toc224893978)

[Supplementary Table 8. Subgroup analysis among children breastfed for at least six months: cohort-specific and pooled estimates for the association between timing of any complementary food introduction and inflammatory bowel disease.	16](#_Toc224893979)

[Supplementary Table 9. Pooled estimates for the timing of complementary food introduction and the risk of childhood-onset inflammatory bowel disease (<18 years).	17](#_Toc224893980)

[Supplementary Table 10. Data description of food introduction by food groups in ABIS and MoBa cohorts.	18](#_Toc224893981)

[Supplementary Table 11. Pooled estimates for the association between the timing of individual food group introduction and inflammatory bowel disease.	20](#_Toc224893982)

[Supplementary Table 12. Timing of any complementary food introduction and identified food group patterns and the risk of inflammatory bowel disease in ABIS and MoBa cohorts.	22](#_Toc224893983)

[Supplementary Table 13. Timing of any complementary food introduction and identified food group patterns and the risk of Crohn’s disease in ABIS and MoBa cohorts.	23](#_Toc224893984)

[Supplementary Table 14. Timing of any complementary food introduction and identified patterns and the risk of ulcerative colitis in ABIS and MoBa cohorts.	24](#_Toc224893985)

[Supplementary Figures and Materials.	25](#_Toc224893986)

[Supplementary Figure 1. Data collection description	25](#_Toc224893987)

[Description of data collection in the	25](#_Toc224893988)

[Supplementary Figure 2. Directed Acyclic Graph (DAG)	26](#_Toc224893989)

[Directed acyclic graph (DAG) describing the analysis of the timing of complementary food introduction and risk of inflammatory bowel disease (IBD). The DAG was created at www.dagitty.net.	26](#_Toc224893990)

[Supplementary Material 1. Descriptives of the Latent Class Analysis (LCA) and identified patterns of food group introduction performed in the ABIS cohort and applied to the MoBa cohort.	27](#_Toc224893991)

[Supplementary Material 2. Covariate data description	29](#_Toc224893992)

[REFERENCES	30](#_Toc224893993)

# Supplementary Tables.

## Supplementary Table 1. Earlier publications on complementary or solid food introduction and risk of inflammatory bowel disease.

| *Last search was conducted on February 12, 2025* | *Search terms:*   1) Infant OR baby OR babies OR early life AND 2) Early feeding OR weaning OR food introduction OR feeding OR complementary food* OR solid food* AND 3) Inflammatory bowel disease OR Chron's disease OR ulcerative colitis | | | | |
| --- | --- | --- | --- | --- | --- |
| **Authors,** year; country; PMID | ***Title* (study design)** | **Study population** | **Exposures** | **Outcomes** | **Results** |
| **Gilat et al.,** 1987; International (9 countries); PMID: 3685876^1^ | *Childhood factors in ulcerative colitis and Crohn's disease. An international cooperative study*  (Case-control) | 197 UC cases  302 CD cases  (499 IBD)  Two age- and sex-matched controls for each patient. | Age of starting mixed feedings (mixed feeding not specified).  Age at study entry <25 years.  Data gathering through questionnaires 1981–1984. | UC or CD  Disease onset before age 20 years, age at the time of study less than 25 years | No differences between patients and controls in the age of starting mixed feedings in infancy. Relative risks were not presented. |
| **Koletzko et al.,** 1989; Canada;  PMID: 2503151^2^ | *Role of infant feeding practices in development of Crohn's disease in childhood*  (Case-control) | 114 patients  180 unaffected siblings | Solid food introduction.  A questionnaire sent to families with at least 1 child aged <18 years with CD (median age 15.7 years). | CD <18 years | Age at introduction of solid foods did not differ between CD patients and controls (siblings). Odds ratios (OR) were not presented. |
| **Koletzko et al.,** 1991; Canada; PMID: 1855043^3^ | *Infant feeding practices and ulcerative colitis in childhood*  (Case-control) | 93 patients  138 unaffected siblings | Solid food introduction.  A questionnaire sent to families with at least 1 child with UC (median age 15.0 years). | UC (children) | Age at introduction of solid foods did not differ between UC patients and controls (siblings). OR not presented. |
| **Baron et al.,** 2005; France; PMID: 15710983^4^ | *Environmental risk factors in paediatric inflammatory bowel diseases: a population based case control study*  (Case-control) | 222 CD cases  60 UC cases  One matched control per case (by sex, age, and geographical location). | Infant diet (not specified).  Collected by interviews with the subject (<17 years of age at diagnosis) and the mother in the home. | CD or UC  (diagnosis between 1 January 1988 and 31 December 1997) | No significant difference in the age of introduction of flour, meat, and vegetables, OR not presented. |
| **Garcia-Larsen et al.,** 2018; PMID: 29489823^5^ | *Diet during pregnancy and infancy and risk of allergic or autoimmune disease: A systematic review and meta-analysis*  (Systematic Review and Meta-Analysis) | Observational studies between January 1946 and July 2013. Intervention studies until December 2017. | Total breastfeeding, exclusive breastfeeding or timing of solid food introduction, from 0–12 months of age. | Allergic or autoimmune diseases, among others. | No association found for “the other autoimmune diseases” (including inflammatory bowel disease, 13 studies*).  *On total breastfeeding, exclusive breastfeeding **or** timing of solid food introduction. |
| **Soranio et al.,** 2023; international collaboration;  PMID: 36704902^6^ | *Complementary and Allergenic Food Introduction in Infants:* *An Umbrella Review*  (Umbrella Review) | Systematic reviews and Meta-analyses.  Human participants of any age. | Complementary food introduction (age); any solid, semisolid, or soft food, from 0–12 months of age. | Autoimmune diseases (including IBD). | Insufficient evidence for an association between the timing of complementary or allergenic food introduction and the risk of gastrointestinal diseases in general. |
| **Fantodji et al.,** 2025; Canada; PMID: 39379227^7^ | *Early life exposures and risk of inflammatory bowel disease: A nested case-control study in Quebec, Canada*  (Case-control) | 1 212 CD cases  570 UC cases  946 controls  from the Quebec Birth Cohort on Immunity and Health, born between 1970–1974. | Age at introduction of solid food; by three categories:  <3 months,  3–6 months,  >6 months.  Reported by web/telephone questionnaire, study conducted 2021. | CD or UC | Introduction of solid foods <3 months compared to introduction >6 months:  Adjusted OR CD = 0.91; 95% CI 0.68–1.20  Adjusted OR UC = 1.01; 95% CI 0.71–1.44  Introduction of solid foods at 3–6 months compared to introduction >6 months:  Adjusted OR CD = 1.23; 95% CI: 0.96–1.56  Adjusted OR UC = 1.04; 95% CI 0.77–1.41 |
| CD, Crohn's disease; IBD, inflammatory bowel disease; UC, ulcerative colitis | | | | | |

## Supplementary Table 2. Exposure definitions of measures of food introduction in ABIS and MoBa cohorts, and the harmonization of these variables.

| **Exposure** | **Description** | **MoBa** | **ABIS** |
| --- | --- | --- | --- |
| **Timing of any food introduction** | Age (in months) at which the complementary food was first introduced into the child's diet. The lowest age applies in cases where multiple data values were available. Examined by age [months] – categorized into the following age groups;  **<4 months**  **4-5 months**  **≥6 months (ref)**  ***MoBa:***  **The lowest written age applied:**  *Six-month questionnaire:*  Question 18: The lowest age reported for any food introduction (options 1–17).  *Due to the timing of questionnaire administration in MoBa, the oldest age at first food introduction was restricted to 9 months.*  ***ABIS:***  Primarily:  **The lowest reported age (calculated from date of birth until reported date) of food diary data:**  *(Questions 1 and 2 concern breastfeeding and formula milk)*  3. Follow-up formula/gruel with gluten (or if missing: 29, repeated questions on gruel introduction)  4. Follow-up formula/gruel without gluten  5. Soy – Follow-up formula/gruel (or if missing: 32, repeated questions on soy-gruel introduction)  6. Wholegrain Follow-up formula/gruel  7. Corn – Follow-up formula/gruel  8. Porridge with gluten (wheat/rye) (or if missing: 30, repeated questions on porridge introduction)  9. Oats Porridge  10. Rice Porridge  11. Semolina Porridge  12. Cheese  13. Milk (cow) (or if missing: 31, repeated questions on milk introduction)  14. Sour milk/yoghurt  15. Bread with gluten (wheat/rye) (or if missing: 33, repeated questions on bread introduction)  16. Pasta/spaghetti (or if missing: 34, repeated questions on pasta introduction)  17. Potato  18. Rice  19. Fruit, berries  20. Lemonade, soda  21. Sugar  22. Vegetables  23. Meat  24. Fish (or if missing: 35, repeated questions on fish introduction)  Secondarily:  **If diary data was missing, the lowest written age applied for any of the following:**  *1year questionnaire:*  Q9 (food with gluten)  Q72 (follow-up formula/gruel)  Q76 (porridge)  Q86 (any food)  Q87b (canned food, if Q87=“yes”)  Q98b (yogurt/sour milk) | **Questionnaire around six months of age (Q18)**  How often does your child eat the following food at the moment, and how old was your child when you started giving him/her this food?  (Never / 1 – 3 times a week / 4 – 6 times a week / at least once a day + age in months)  *Instant porridge*   1. Rice porridge, maize porridge 2. Oatmeal, different types 3. Wheat porridge, all types, rusk porridge   *Home-made porridge using:*   4. Wheat flour, rusk, semolina, oats 5. Iron-enriched wheat flour 6. Helios baby flour 7. Millet   *Processed dinner in a jar:*   8. Vegetables 9. Vegetables and meat   *Home-made dinner:*   10. Potato/vegetable puree 11. Meat and vegetable/potato 12. Fish and vegetable/potato 13. Other types of home-made dinner   *Snack/dessert:*   14. Home-made fruit puree 15. Fruit/berry puree in a jar 16. Rusks/biscuits/bread 17. Other | **Food diary of food introduction, from birth until one year age:**  **Date for when the child starts with the following foods (YYMMDD):**  3. Follow-up formula/gruel with gluten^a^  4. Follow-up formula/gruel without gluten^a^  5. Soy – Follow-up formula/gruel  6. Wholegrain Follow-up formula/gruel^a^  7. Corn – Follow-up formula/gruel  8. Porridge with gluten (wheat/rye)  9. Oats porridge  10. Rice Porridge  11. Semolina Porridge  12. Cheese  13. Milk (cow)  14. Sour milk/yoghurt  15. Bread with gluten (wheat/rye)  16. Pasta/spaghetti  17. Potato  18. Rice  19. Fruit, berries  20. Lemonade, soda  21. Sugar  22. Vegetables  23. Meat  24. Fish  **Questionnaire around one year age (Q9)**  When did the child get gluten (e.g., in milk food, pasta, macaroni, bread, etc.) for the first time?   1. 1 month 2. 2 months 3. 3 months 4. 4 months 5. 5 months 6. 6 months 7. 7months 8. 8 months 9. 9 months and later   **1 year of age (Q76)**  At what age did your child receive follow-up formula/gruel for the first time?^a^   1. 1 month 2. 2 months 3. 3 months 4. 4 months 5. 5 months 6. 6 months 7. 7months 8. 8 months 9. 9 months and later   **1 year of age (Q76)**  At what age did your child receive porridge for the first time?   1. 1 month 2. 2 months 3. 3 months 4. 4 months 5. 5 months 6. 6 months 7. 7months 8. 8 months 9. 9 months and later   **1 year of age (Q86)**  At what age did your child taste a food for the first time?   1. 2 months 2. 3 months 3. 4 months 4. 5 months 5. 6 months 6. 7 months 7. 8 months 8. 9 months 9. 10 months and later   **1 year of age (Q87)**  Have children been fed canned baby food?   1. Yes 2. No   **1 year of age (Q87b)**  If yes Q87, at what age did your child receive canned food for the first time   1. 2 months 2. 3 months 3. 4 months 4. 5 months 5. 6 months 6. 7 months 7. 8 months 8. 9 months 9. 10 months and later   **1 year of age (Q98b)**  At what age (months) did your child receive yogurt/sour milk for the first time?   1. 1 month 2. 2 months 3. 3 months 4. 4 months 5. 5. months 6. 6 months 7. 7 months 8. 8 months 9. 9 months and later |
| **Exposure** | **Description** | **MoBa** | **ABIS** |
| **Food introduction timing by LCA pattern** | Age (months) for when different food groups were introduced. The lowest age applies if multiple data values are available.  Examined by age [months] – categorized into age groups;  **<4 months**  **4-5 months**  **≥6 months (ref)**  *Except for dairy:*  **<6 months**  **6-8 months (ref)**  **≥9 months**  **Any cereal (w/wo gluten)**  ***MoBa***  1. Rice porridge, maize porridge  2. Oatmeal, different types  3. Wheat porridge, all types, rusk porridge  4. Wheat flour, rusk, semolina, oats  5. Iron-enriched wheat flour  6. Helios baby flour  7. Millet  16. Rusks/biscuits/bread  ***ABIS***  Diary  3. Follow-up formula/gruel with gluten (or if missing: 29, repeated questions on gruel introduction)  4. Follow-up formula/gruel without gluten  6. Wholegrain Follow-up formula/gruel  7. Corn – Follow-up formula/gruel  8. Porridge with gluten (wheat/rye) (or if missing: 30, repeated questions on porridge introduction)  9. Oats Porridge  10. Rice Porridge  11. Semolina Porridge  15. Bread with gluten (wheat/rye) (or if missing: 33, repeated questions on bread introduction)  16. Pasta/spaghetti (or if missing: 34, repeated questions on pasta introduction  18. Rice  **Cereal with gluten**  ***MoBa***  3. Wheat porridge, all types, rusk porridge  4. Wheat flour, rusk, semolina, oats  5. Iron-enriched wheat flour  6. Helios baby flour  16. Rusks/biscuits/bread  ***ABIS***  Diary  3. Follow-up formula/gruel with gluten  6. Wholegrain Follow-up formula/gruel  8. Porridge with gluten (wheat/rye)  11. Semolina Porridge  15. Bread with gluten (wheat/rye)  16. Pasta/spaghetti (or if missing: 34, repeated questions on pasta introduction)  **Fruits and berries**  ***MoBa***  14. Home-made fruit puree  15. Fruit/berry puree in jar  ***ABIS***  Diary  19. Fruit, berries  **Vegetable and potatoes**  ***MoBa***  8. Vegetables  9. Vegetables and meat  10. Potato/vegetable puree  11. Meat and vegetable/potato  12. Fish and vegetable/potato  ***ABIS***  Diary  17. Potato  22. Vegetables  **Meat**  ***MoBa***  9. Vegetables and meat  11. Meat and vegetable/potato  ***ABIS***  Diary  23. Meat  **Fish**  ***MoBa***  12. Fish and vegetable/potato  ***ABIS***  Diary  24. Fish (or if missing: 35, repeated questions on fish introduction)  **Dairy**  ***MoBa***  *Introduction before 6 months (Q17) if any intake of:*  3. Normal sweet milk, any type  4. Sour milk (yogurt, buttermilk, etc.)  5. Organic milk products (milk, yogurt)  *If reporting “Never/seldom” of dairy intake in the 6 months questionnaire – later introduction was assumed. Age by pre-defined age groups were retrieved from the 18 months questionnaire (Q1) by ANY of the following food items:*  4. Whole milk (sweet)  5. Low-fat milk normal (sweet)  6. Extra low-fat milk (sweet)  7. Skimmed milk (sweet)  8. Yogurt with active Lactobacillus, all types  9. Other yogurt  10. Other types of sour milk  ***ABIS***  Diary  12. Cheese  13. Milk (cow) (or if missing: 31, repeated questions on milk introduction)  14. Sour milk/yoghurt | **6 months of age (Q18)**  How often does your child eat the following food at the moment, and how old was your child when you started giving him/her this food?  (age in months)  *Instant porridge*   1. Rice porridge, maize porridge 2. Oatmeal, different types 3. Wheat porridge, all types, rusk porridge   *Home-made porridge using:*   4. Wheat flour, rusk, semolina, oats 5. Iron-enriched wheat flour 6. Helios baby flour 7. Millet   *Processed dinner in a jar:*   8. Vegetables 9. Vegetables and meat   *Home-made dinner:*   10. Potato/vegetable puree 11. Meat and vegetable/potato 12. Fish and vegetable/potato   *Snack/dessert:*   13. Home-made fruit puree 14. Fruit/berry puree in a jar 15. Rusks/biscuits/bread   **6 months of age (Q17)**  **How often do you give your child the following to drink at the moment?**  *…*  *3. Normal sweet milk, any type*  *4. Sour milk (yogurt, buttermilk, etc.)*  *5. Organic milk products (milk, yogurt)*  *…  Response options:*  **1) Never/seldom**  2) 1-3 times a week  3) 4-6 times a week  4) At least once a day  *If reporting Never/seldom, data was retrieved from the 18 months questionnaire:*  **18 months of age (Q1)**  What type of milk has your baby been given since he/she was 6 months old?  (You can enter more than one cross.)  *Child’s age in months:*   - 6-8 months - 9-11 months - 12-14 months   *(response option “15-18 months” was not considered due to data availability in ABIS)* | **Food diary 1 year:**  **Date for when the child starts with the following foods (YYMMDD):**  3. Follow-up formula/gruel with gluten^a^  4. Follow-up formula/gruel without gluten^a^  5. Soy – Follow-up formula/gruel  6. Wholegrain Follow-up formula/gruel^a^  7. Corn – Follow-up formula/gruel  8. Porridge with gluten (wheat/rye)  9. Oats porridge  10. Rice Porridge  11. Semolina Porridge  12. Cheese  13. Milk (cow)  14. Sour milk/yoghurt  15. Bread with gluten (wheat/rye)  16. Pasta/spaghetti  17. Potato  18. Rice  19. Fruit, berries  20. Lemonade, soda  21. Sugar  22. Vegetables  23. Meat  24. Fish |
| ^a^In Sweden, the follow-on formula/gruel (recommended from six months of age) is typically based on cereal and milk prepared as a powder to mix with water or as a ready-to-drink liquid. ABIS, All Babies in Southeast Sweden study; LCA, Latent Class Analysis; MoBa, The Norwegian Mother, Father, and Child Cohort Study. | | | |

## Supplementary Table 3. Register-based definition of inflammatory bowel disease.

| Outcome definitions (≥2 entries of relevant ICD-10 codes for IBD)^1^ | |
| --- | --- |
| **IBD** = UC + CD, mixed UC-CD, or K52.3 | IBD diagnosis is defined by ≥ 2 inpatient or non-primary outpatient care visits listing an IBD diagnosis, which has a high positive predictive value in Sweden and Norway [8, 9]. The first recorded diagnosis defined the onset of disease [10]. The most recently recorded ICD-10 code defined the diagnosis for patients shifting between CD and UC, but with only one of the diagnoses during the last five years. Patients with a mix of codes for UC, CD, or indeterminate colitis during follow-up were classified as (“any, non-specific”) IBD. |
| **CD** = K50 |  |
| **UC** = K51 |  |
| ^a^Definition of IBD was largely consistent with the register-based IBD definition used by Everhov AH et al. [11]. CD, Crohn's disease; IBD, inflammatory bowel disease; ICD-10, International Classification of Diseases; UC, ulcerative colitis. | |

## Supplementary Table 4. Characteristics of participants by IBD, CD & UC.

|  | ABIS | | | |  | MoBa | | | |
| --- | --- | --- | --- | --- | --- | --- | --- | --- | --- |
|  | All  N = 11 947 | IBD^c^  n = 124 | CD  n = 45 | UC  n = 65 |  | All  N = 82 291 | IBD^c^  n = 276 | CD  n = 117 | UC  n = 78 |
| Sex (%) |  |  |  |  |  |  |  |  |  |
| Male | 6 209 (52.0) | 62 (50.0) | 21 (46.7) | 34 (52.3) |  | 42 128 (51.2) | 161 (58.3) | 70 (59.8) | 35 (44.9) |
| Female | 5 738 (48.0) | 62 (50.0) | 24 (53.3) | 31 (47.7) |  | 40 163 (48.8) | 115 (41.7) | 47 (40.2) | 43 (55.1) |
| Age at the end of follow-up^a^ (years) |  |  |  |  |  |  |  |  |  |
| Mean (SD) | 25.2 (1.1) | 18.8 (4.0) | 17.8 (3.9) | 19.3 (4.0) |  | 16.5 (2.2) | 12.8 (3.7) | 12.9 (3.3) | 13.6 (3.7) |
| Median  (interquartile range) | 25.3  (24.8, 25.8) | 19.2  (16.3, 22.1) | 18.4  (15.6, 20.6) | 19.8  (17.3, 22.6) |  | 16.2  (14.8, 18.2) | 13.2  (10.7, 15.6) | 12.8  (10.8, 15.1) | 14.5  (11.6, 16.5) |
| Follow-up time (years)^b^ |  |  |  |  |  |  |  |  |  |
| Mean (SD) | 24.2 (1.1) | 17.8 (4.0) | 16.8 (3.9) | 18.3 (4.0) |  | 15.5 (2.2) | 11.8 (3.7) | 11.9 (3.3) | 12.6 (3.7) |
| Median  (interquartile range) | 24.3  (23.8, 24.8) | 18.2  (15.3, 21.1) | 17.4  (14.6, 19.6) | 18.8  (16.3, 21.6) |  | 15.2  (13.7, 17.2) | 12.2  (9.7, 14.6) | 11.8  (9.7, 14.1) | 13.5  (10.6, 15.5) |
| Calendar year of birth (%) |  |  |  |  |  |  |  |  |  |
| 1997 | 1 329 (11.1) | 12 (9.7) | 3 (6.7) | 8 (12.3) |  | - | - | - | - |
| 1998 | 6 311 (52.8) | 74 (59.7) | 29 (64.4) | 36 (55.4) |  | - | - | - | - |
| 1999 | 4 307 (36.1) | 38 (30.6) | 13 (28.9) | 21 (32.3) |  | 23 (<0.1) | 0 (0.0) | 0 (0.0) | 0 (0.0) |
| 2000 | - | - | - | - |  | 1 604 (1.9) | 6 (2.2) | 2 (1.7) | 2 (2.6) |
| 2001 | - | - | - | - |  | 3 213 (3.9) | 22 (8.0) | 4 (3.4) | 9 (11.5) |
| 2002 | - | - | - | - |  | 6 791 (8.3) | 35 (12.7) | 15 (12.8) | 8 (10.3) |
| 2003 | - | - | - | - |  | 9 963 (12.1) | 44 (15.9) | 18 (15.4) | 15 (19.2) |
| 2004 | - | - | - | - |  | 10 609 (12.9) | 42 (15.2) | 19 (16.2) | 12 (15.4) |
| 2005 | - | - | - | - |  | 12 039 (14.7) | 35 (12.7) | 13 (11.1) | 12 (15.4) |
| 2006 | - | - | - | - |  | 13 350 (16.2) | 32 (11.6) | 15 (12.8) | 7 (9.0) |
| 2007 | - | - | - | - |  | 12 093 (14.7) | 24 (8.7) | 12 (10.3) | 6 (7.7) |
| 2008 | - | - | - | - |  | 10 084 (12.3) | 33 (12.0) | 18 (15.4) | 6 (7.7) |
| 2009 | - | - | - | - |  | 2 522 (3.1) | 3 (1.1) | 1 (0.9) | 1 (1.3) |
| Food introduction timing by LCA pattern (%) |  |  |  |  |  |  |  |  |  |
| Early introducers | 375 (3.1) | 5 (4.0) | 2 (4.4) | 1 (1.5) |  | 2 379 (2.9) | 10 (3.6) | 6 (5.1) | 1 (1.3) |
| Mid introducers | 4 046 (33.9) | 43 (34.7) | 22 (48.9) | 15 (23.1) |  | 18 927 (23.0) | 68 (24.6) | 27 (23.1) | 21 (26.9) |
| Mid-Late introducers | 2 611 (21.9) | 26 (21.0) | 4 (8.9) | 20 (30.8) |  | 44 004 (53.5) | 139 (50.4) | 62 (53.0) | 40 (51.3) |
| Late introducers | 2 352 (19.7) | 24 (19.4) | 9 (20.0) | 14 (21.5) |  | 16 936 (20.6) | 59 (21.4) | 22 (18.8) | 16 (20.5) |
| Missing^d^ | 2 563 (21.5) | 26 (21.0) | 8 (17.8) | 15 (23.1) |  | 45 (<0.1) | 0 (0.0) | 0 (0.0) | 0 (0.0) |
| Start of formula feeding (%) |  |  |  |  |  |  |  |  |  |
| <1 month | 1 540 (12.9) | 16 (12.9) | 3 (6.7) | 10 (15.4) |  | 17 572 (21.4) | 55 (19.9) | 25 (21.4) | 15 (19.2) |
| 1–3 months | 1 893 (15.8) | 23 (18.5) | 10 (22.2) | 9 (13.8) |  | 12 345 (15.0) | 40 (14.5) | 21 (17.9) | 9 (11.5) |
| ≥4 months | 1 384 (11.6) | 15 (12.1) | 8 (17.8) | 5 (7.7) |  | 24 277 (29.5) | 78 (28.3) | 38 (32.5) | 21 (26.9) |
| Missing | 7 130 (59.7) | 70 (56.5) | 24 (53.3) | 41 (63.1) |  | 28 097 (34.1) | 103 (37.3) | 33 (28.2) | 33 (42.3) |
| Any breastfeeding duration^e^ (%) |  |  |  |  |  |  |  |  |  |
| 0–<4 months | 1 468 (12.3) | 15 (12.1) | 7 (15.6) | 5 (7.7) |  | 10 638 (12.9) | 34 (12.3) | 16 (13.7) | 7 (9.0) |
| 4–<6 months | 1 113 (9.3) | 14 (11.3) | 5 (11.1) | 8 (12.3) |  | 5 903 (7.2) | 21 (7.6) | 10 (8.5) | 3 (3.8) |
| ≥6 months | 8 397 (70.3) | 77 (62.1) | 28 (62.2) | 40 (61.5) |  | 65 750 (79.9) | 221 (80.1) | 91 (77.8) | 68 (87.2) |
| Missing | 969 (8.1) | 18 (14.5) | 5 (11.1) | 12 (18.5) |  | 0 (0.0) | 0 (0.0) | 0 (0.0) | 0 (0.0) |
| Maternal age at delivery^f^ (%) |  |  |  |  |  |  |  |  |  |
| <25 years | 1 689 (14.1) | 15 (12.1) | 6 (13.3) | 7 (10.8) |  | 8 326 (10.1) | 28 (10.1) | 10 (8.5) | 8 (10.3) |
| 25-34 years | 8 586 (71.9) | 93 (75.0) | 31 (68.9) | 50 (76.9) |  | 59 624 (72.5) | 202 (73.2) | 88 (75.2) | 57 (73.1) |
| ≥35 years | 1 477 (12.4) | 13 (10.5) | 6 (13.3) | 7 (10.8) |  | 14 298 (17.4) | 46 (16.7) | 19 (16.2) | 13 (16.7) |
| Missing | 195 (1.6) | 3 (2.4) | 2 (4.4) | 1 (1.5) |  | 43 (0.1) | 0 (0.0) | 0 (0.0) | 0 (0.0) |
| Parental IBD (%) |  |  |  |  |  |  |  |  |  |
| No | 11 795 (98.7) | 117 (94.4) | 42 (93.3) | 62 (95.4) |  | 80 391 (97.7) | 268 (97.1) | 113 (96.6) | 76 (97.4) |
| Yes | 152 (1.3) | 7 (5.6) | 3 (6.7) | 3 (4.6) |  | 1 900 (2.3) | 8 (2.9) | 4 (3.4) | 2 (2.6) |
| Missing | 0 (0.0) | 0 (0.0) | 0 (0.0) | 0 (0.0) |  | 0 (0.0) | 0 (0.0) | 0 (0.0) | 0 (0.0) |
| Parental origin^g^ (%) |  |  |  |  |  |  |  |  |  |
| Norwegian/Swedish | 10 568 (88.5) | 108 (87.1) | 41 (91.1) | 55 (84.6) |  | 71 077 (86.4) | 237 (85.9) | 104 (88.9) | 64 (82.1) |
| Other | 1 123 (9.4) | 15 (12.1) | 4 (8.9) | 9 (13.8) |  | 7 771 (9.4) | 26 (9.4) | 10 (8.5) | 7 (9.0) |
| Missing | 256 (2.1) | 1 (0.8) | 0 (0.0) | 1 (1.5) |  | 3 443 (4.2) | 13 (4.7) | 3 (0.3) | 7 (9.0) |
| Maternal education level^h^ (%) |  |  |  |  |  |  |  |  |  |
| Low | 840 (7.0) | 15 (12.1) | 6 (13.3) | 8 (12.3) |  | 5 577 (6.8) | 26 (9.4) | 14 (12.0) | 4 (5.1) |
| Medium | 6 496 (54.4) | 69 (55.6) | 26 (57.8) | 36 (55.4) |  | 23 576 (28.8) | 80 (29.0) | 30 (25.6) | 24 (30.8) |
| High | 4 344 (36.4) | 39 (31.5) | 13 (28.9) | 20 (30.8) |  | 52 821 (64.2) | 168 (60.9) | 73 (62.4) | 50 (64.1) |
| Missing | 267 (2.2) | 1 (0.8) | 0 (0.0) | 1 (1.5) |  | 317 (0.4) | 2 (0.7) | 0 (0.0) | 0 (0.0) |
| ^a^Age at the end of the study equals age at diagnosis for all IBD, CD, and UC events. ^b^Follow-up started at one year. ^c^Includes IBD-unclassified (IBD-U). ^d^Missingness in ABIS for patterns of food group introduction reflects a higher proportion of participants with only data on overall complementary food introduction but not on individual food groups. ^e^Partial or exclusive breastfeeding duration. ^f^Categories were merged for the table presentation, and the following categories were used in the analyses: 15-19/20-24/25-29/30-34/35-39/40-44 years. ^g^ABIS: other country if any parent was born in another country than Sweden; MoBa: any parent having a different native language than Norwegian. ^h^High education level: ≥13 years of education, medium education level: 12 years of education, and low education level: 9 years in ABIS; 9-11 years in MoBa. ABIS, All Babies in Southeast Sweden study; CD, Crohn's disease; IBD, inflammatory bowel disease; LCA, Latent Class Analysis; MoBa, The Norwegian Mother, Father, and Child Cohort Study; SD, standard deviation; UC, ulcerative colitis. | | | | | | | | | |

## Supplementary Table 5. Person-years of follow-up, crude incidence rates, and age-and-sex standardized incidence rates for inflammatory bowel disease in ABIS and MoBa cohorts.

|  | N events  (% of total study population) | Person-years of follow-up | Incidence per 100,000 PYR (95%CI) | Age- and sex-standardized incidence per 100,000 PYR^a^ (95%CI) |
| --- | --- | --- | --- | --- |
| ABIS^b^ |  |  |  |  |
| IBD^c^ | 124 (1.0) | 289 312 | 42.9 (35.9, 51.1) | 23.4 (15.5, 35.4) |
| CD | 45 (0.4) | 289 783 | 15.5 (11.6, 20.8) | 11.0 (6.7, 18.2) |
| UC | 65 (0.5) | 289 729 | 22.4 (17.6, 28.6) | 10.2 (6.5, 16.2) |
| MoBa^b^ |  |  |  |  |
| IBD^c^ | 276 (0.3) | 1 273 038 | 21.7 (19.2, 24.4) | - |
| CD | 117 (0.1) | 1 273 765 | 9.2 (7.6, 11.0) | - |
| UC | 78 (0.1) | 1 273 926 | 6.1 (4.8, 7.6) | - |
| ^a^Age- and sex- standardized according to the incidence rate in the MoBa population and applied to the ABIS population. ^b^Mean age at the end of follow-up was 25.2 years in ABIS and 16.5 years in MoBa. ^c^IBD also includes IBD-unclassified. ABIS, All Babies in Southeast Sweden study; CD, Crohn's disease; CI, confidence interval; IBD, inflammatory bowel disease; MoBa, The Norwegian Mother, Father and Child Cohort Study; PYR, person-years; UC, ulcerative colitis. | | | | |

## Supplementary Table 6. Characteristics of those with valid data and missing data on complementary food introduction in the ABIS and MoBa cohorts.

|  | ABIS | |  | MoBa | |  |
| --- | --- | --- | --- | --- | --- | --- |
|  | Valid data on complementary food introduction | Missing data on complementary food introduction |  | Valid data on complementary food introduction | Missing data on complementary food introduction | |
|  | n = 11 947 | n = 4 276 |  | n = 82 291 | n = 18 951 | |
| IBD incidence per 100,000 person-years (95% CI) | 42.9  (35.9, 51.1) | 27.1^a^ (18.0, 39.2) |  | 21.7  (19.2, 24.4) | 21.1  (16.1, 27.1) | |
| Smoking in pregnancy (%) | | | | | | |
| No | 10 596 (88.7) | 3 487 (81.5) |  | 73 824 (89.7) | 16 304 (85.9) | |
| Yes | 1 088 (9.1) | 672 (15.7) |  | 7 134 (8.7) | 2 347 (12.4) | |
| Missing | 263 (2.2) | 117 (2.7) |  | 1 333 (1.6) | 328 (1.7) | |
| Maternal education level^b^ (%) | | | | | | |
| Low | 840 (7.0) | 518 (12.1) |  | 5 577 (6.8) | 2 452 (12.9) | |
| Medium | 6 496 (54.4) | 2 317 (54.2) |  | 23 576 (28.8) | 6 159 (32.5) | |
| High | 4 344 (36.4) | 1 325 (31.0) |  | 52 821 (64.2) | 10 176 (53.6) | |
| Missing | 267 (2.2) | 116 (2.7) |  | 317 (0.4) | 192 (1.0) | |
| Maternal age at delivery (%) | | | | | | |
| <25 years | 1 689 (14.1) | 862 (20.2) |  | 8 326 (10.1) | 2 745 (14.5) | |
| 25-34 years | 8 586 (71.9) | 2 854 (66.7) |  | 59 624 (72.5) | 12 807 (67.5) | |
| ≥35 years | 1 477 (12.4) | 495 (11.6) |  | 14 298 (17.4) | 3 416 (18.0) | |
| Missing | 195 (1.6) | 65 (1.5) |  | 43 (0.1) | 11 (0.1) | |
| ^a^Among those missing data in ABIS, 28 individuals (0.7% ) had an IBD diagnosis over 103,159 person-years of follow-up, compared to those with valid data where 124 individuals (1.0%) developed IBD over 289,3132 person-years of follow-up. ^b^High education level equaled ≥13 years of education, medium education level 12 years of education, and low education level 9-11 years of education (9 years in ABIS; 9-11 years in MoBa). ABIS, All Babies in Southeast Sweden study; CI, confidence interval; MoBa, The Norwegian Mother, Father, and Child Cohort Study. | | | | | |  |

## Supplementary Table 7. Sensitivity analyses: pooled estimates for the timing of complementary food introduction and inflammatory bowel disease risk, including adjustment for breastfeeding duration and start of formula feeding, or perinatal factors.

| Pooled analysis | Crude analyses | | |  | Sensitivity analysis adjusted for  infant feeding practices^a^ | | |  | Sensitivity analysis adjusted for  perinatal factors^b^ | | | |
| --- | --- | --- | --- | --- | --- | --- | --- | --- | --- | --- | --- | --- |
| Characteristic | N | Event n | HR (95% CI) |  | N | Event n | HR (95% CI) |  | N | | Event n | HR (95% CI) |
| Timing of any food introduction | |  |  |  |  |  |  |  |  | |  |  |
| *Before 4 months* | 6 199 | 36 | 1.08 (0.71, 1.63) |  | 4 681 | 26 | 1.16 (0.64, 2.08) |  | 4 396 | 27 | | 0.99 (0.60, 1.66) |
| *4-5 months* | 71 905 | 299 | 0.87 (0.62, 2.10) |  | 45 134 | 160 | 0.82 (0.52, 1.27) |  | 54 382 | 225 | | 0.86 (0.52, 1.42) |
| *6 months or later* | 14 790 | 64 | (Reference) |  | 5 688 | 23 | (Reference) |  | 11 240 | 51 | | (Reference) |
| Food introduction timing by LCA pattern | |  |  |  |  |  |  |  |  | |  |  |
| *Early introducers* | 2 754 | 15 | 1.24 (0.73, 2.12) |  | 2 207 | 11 | 1.34 (0.71, 2.53) |  | 1 888 | | 10 | 1.13 (0.58, 2.20) |
| *Mid introducers* | 22 973 | 111 | 1.05 (0.82, 1.34) |  | 15 189 | 76 | 1.30 (0.94, 1.79) |  | 15 199 | | 76 | 1.03 (0.76, 1.39) |
| *Mid-Late introducers* | 46 614 | 165 | (Reference) |  | 30 492 | 92 | (Reference) |  | 37 109 | | 131 | (Reference) |
| *Late introducers* | 19 288 | 83 | 1.07 (0.82, 1.40) |  | 8 073 | 30 | 1.09 (0.71, 1.66) |  | 14 867 | | 63 | 1.03 (0.63, 1.71) |
| ^a^Adjusted for child’s sex and birth year, maternal age and education level, parental inflammatory bowel disease and origin, any breastfeeding duration, and the start of formula feeding. ^b^Adjusted for child’s sex and birth year, maternal age and education level, parental inflammatory bowel disease and origin, maternal smoking during pregnancy, birth weight, delivery mode, gestational age, diet diversity at one year age, and antibiotic use by one year age. ABIS, All Babies in Southeast Sweden study; CI, Confidence Interval; HR, Hazard Ratio; LCA, Latent Class Analysis; MoBa, The Norwegian Mother, Father, and Child Cohort Study. | | | | | | | | | | | | |

## Supplementary Table 8. Subgroup analysis among children breastfed for at least six months: cohort-specific and pooled estimates for the association between timing of any complementary food introduction and inflammatory bowel disease.

| *Stratified by any breastfeeding duration for six months or longer* | Crude analyses | | |  | Adjusted analyses including child’s  sex, birth year, and parental factors^a^ | | |
| --- | --- | --- | --- | --- | --- | --- | --- |
| Characteristic | N | Event n | HR (95% CI) |  | N | Event n | HR (95% CI) |
| Timing of any food introduction | |  |  |  |  |  |  |
| ABIS | |  |  |  |  |  |  |
| *Before 4 months* | 570 | 5 | 0.90 (0.30, 2.70) |  | 550 | 5 | 0.82 (0.27, 2.51) |
| *4-5 months* | 6 902 | 63 | 0.94 (0.47, 1.89) |  | 6 662 | 60 | 0.90 (0.44, 1.82) |
| *6 months or later* | 925 | 9 | (Reference) |  | 884 | 9 | (Reference) |
| MoBa | |  |  |  |  |  |  |
| *Before 4 months* | 2 162 | 6 | 0.65 (0.28, 1.52) |  | 2 025 | 6 | 0.65 (0.27, 1.54) |
| *4-5 months* | 49 464 | 164 | 0.85 (0.62, 1.15) |  | 47 116 | 154 | 0.80 (0.58, 1.10) |
| *6 months or later* | 13 055 | 51 | (Reference) |  | 12 591 | 50 | (Reference) |
| Pooled estimates | |  |  |  |  |  |  |
| *Before 4 months* | 2 732 | 11 | 0.74 (0.38, 1.44) |  | 2 575 | 11 | 0.71 (0.36, 1.40) |
| *4-5 months* | 56 366 | 227 | 0.86 (0.65, 1.14) |  | 53 778 | 214 | 0.81 (0.61, 1.09) |
| *6 months or later* | 13 980 | 60 | (Reference) |  | 13 475 | 59 | (Reference) |

^a^Adjusted for child’s sex and birth year, maternal age and education level, parental inflammatory bowel disease, and origin. We were unable to estimate the risk among those with breastfeeding duration <6 months due to no events in the group “complementary food introduction at 6 months or later” in MoBa. ABIS, All Babies in Southeast Sweden study; CI, Confidence Interval; HR, Hazard Ratio; MoBa, The Norwegian Mother, Father, and Child Cohort Study.

## Supplementary Table 9. Pooled estimates for the timing of complementary food introduction and the risk of childhood-onset inflammatory bowel disease (<18 years).

| Pooled analysis | Crude analyses | | |  | Adjusted analyses including child’s  sex, birth year, and parental factors^a^ | | |  | Sensitivity analysis adjusted for  infant feeding practices^b^ | | | |
| --- | --- | --- | --- | --- | --- | --- | --- | --- | --- | --- | --- | --- |
| Characteristic | N | Event n | HR (95% CI) |  | N | Event n | HR (95% CI) |  | N | Event n | HR (95% CI) | |
| Timing of any food introduction | |  |  |  |  |  |  |  |  |  |  | |
| *Before 4 months* | 6 199 | 26 | 1.04 (0.65, 1.65) |  | 5 838 | 25 | 0.91 (0.49, 1.69) |  | 4 681 | 19 | 1.00 (0.37, 2.70) | |
| *4-5 months* | 71 905 | 225 | 0.83 (0.62, 1.11) |  | 68 629 | 213 | 0.79 (0.59, 1.07) |  | 45 134 | 132 | 0.83 (0.52, 1.33) | |
| *6 months or later* | 14 790 | 55 | (Reference) |  | 14 237 | 54 | (Reference) |  | 5 688 | 20 | (Reference) | |
| Food introduction timing by LCA pattern | |  |  |  |  |  |  |  |  |  |  | |
| *Early introducers* | 2 754 | 9 | NA^c^ |  | 2 581 | 8 | NA^c^ |  | 2 207 | 7 | NA^c^ | |
| *Mid introducers* | 22 973 | 81 | 1.15 (0.75, 1.76) |  | 21 585 | 77 | 1.17 (0.78, 1.74) |  | 15 189 | 57 | 1.31 (0.92, 1.85) | |
| *Mid-Late introducers* | 46 614 | 140 | (Reference) |  | 44 825 | 133 | (Reference) |  | 30 492 | 83 | (Reference) | |
| *Late introducers* | 19 288 | 65 | 1.13 (0.79, 1.61) |  | 18 611 | 63 | 1.16 (0.85, 1.57) |  | 8 073 | 24 | 1.06 (0.67, 1.69) | |
| ^a^Adjusted for child’s sex and birth year, maternal age and education level, parental inflammatory bowel disease, and origin. ^b^Adjusted for child’s sex and birth year, maternal age and education level, parental inflammatory bowel disease, origin, any breastfeeding duration, and the start of formula feeding. ^c^Unable to pool cohort-specific estimates due to no events in ABIS. ABIS, All Babies in Southeast Sweden study; CI, Confidence Interval; HR, Hazard Ratio; LCA, Latent Class Analysis; MoBa, The Norwegian Mother, Father, and Child Cohort Study. | | | | | | | | | | | |  |

## Supplementary Table 10. Data description of food introduction by food groups in ABIS and MoBa cohorts.

| ***Food groups*** |  | **ABIS** | | | | |  | **MoBa** | | | | |
| --- | --- | --- | --- | --- | --- | --- | --- | --- | --- | --- | --- | --- |
|  |  | n | % | Valid % | Mean age at introduction, months (SD) | Median age at introduction, months (IQR) |  | n | % | Valid % | Mean age at introduction, months (SD) | Median age at introduction, months (IQR) |
| **Any cereal** |  | N = 9 211 |  |  | 4.9 (1.1) | 5.0 (4.0-6.0) |  | N = 80 150 |  |  | 4.7 (0.9) | 5.0 (4.0-5.0) |
| <4 months |  | 420 | 3.5 | 4.6 |  |  |  | 4 495 | 5.5 | 5.6 |  |  |
| 4-5 months |  | 6 412 | 53.7 | 69.6 |  |  |  | 60 824 | 73.9 | 75.9 |  |  |
| ≥6 months |  | 2 379 | 19.9 | 25.8 |  |  |  | 14 831 | 18.0 | 18.5 |  |  |
| Missing |  | 2 736 | 22.9 |  |  |  |  | 2 141 | 2.6 |  |  |  |
| **Cereal with gluten** |  | N = 9 134 |  |  | 5.2 (1.2) | 5.0 (4.0-6.0) |  | N = 43 004 |  |  | 5.4 (0.8) | 6.0 (5.0-6.0) |
| <4 months |  | 287 | 2.4 | 3.1 |  |  |  | 653 | 0.8 | 1.5 |  |  |
| 4-5 months |  | 5 347 | 44.8 | 58.5 |  |  |  | 19 839 | 24.1 | 46.1 |  |  |
| ≥6 months |  | 3 500 | 29.3 | 38.3 |  |  |  | 22 512 | 27.4 | 52.3 |  |  |
| Missing |  | 2 813 | 23.5 |  |  |  |  | 39 287 | 47.7 |  |  |  |
| **Fruit or berries** |  | N = 8 756 |  |  | 5.2 (1.4) | 5.0 (4.0-6.0) |  | N = 58 037 |  |  | 5.1 (0.8) | 5.0 (4.0-6.0) |
| <4 months |  | 349 | 2.9 | 4.0 |  |  |  | 869 | 1.1 | 1.5 |  |  |
| 4-5 months |  | 5 679 | 47.5 | 64.9 |  |  |  | 36 684 | 44.6 | 63.2 |  |  |
| ≥6 months |  | 2 728 | 22.8 | 31.2 |  |  |  | 20 484 | 24.9 | 35.3 |  |  |
| Missing |  | 3 191 | 26.7 |  |  |  |  | 24 254 | 29.5 |  |  |  |
| **Vegetables or potatoes** |  | N = 9 135 |  |  | 4.6 (1.0) | 4.0 (4.0-5.0) |  | N = 58 214 |  |  | 5.3 (0.8) | 5.0 (5.0-6.0) |
| <4 months |  | 601 | 5.0 | 6.6 |  |  |  | 576 | 0.7 | 1.0 |  |  |
| 4-5 months |  | 7 201 | 60.3 | 78.8 |  |  |  | 30 825 | 37.5 | 53.0 |  |  |
| ≥6 months |  | 1 333 | 11.2 | 14.6 |  |  |  | 26 813 | 32.6 | 46.1 |  |  |
| Missing |  | 2 812 | 23.5 |  |  |  |  | 24 077 | 29.3 |  |  |  |
| **Meat** |  | N = 8 564 |  |  | 5.8 (1.3) | 6.0 (5.0-6.0) |  | N = 38 350 |  |  | 5.6 (0.7) | 6.0 (5.0-6.0) |
| <4 months |  | 30 | 0.3 | 0.4 |  |  |  | 166 | 0.2 | 0.4 |  |  |
| 4-5 months |  | 4 040 | 33.8 | 47.2 |  |  |  | 12 897 | 15.7 | 33.6 |  |  |
| ≥6 months |  | 4 494 | 37.6 | 52.5 |  |  |  | 25 287 | 30.7 | 65.9 |  |  |
| Missing |  | 3 383 | 28.3 |  |  |  |  | 43 941 | 53.4 |  |  |  |
| **Fish** |  | N = 7 028 |  |  | 7.6 (2.2) | 7.0 (6.0-9.0) |  | N = 6 254 |  |  | 5.8 (0.7) | 6.0 (5.0-6.0) |
| <4 months |  | 16 | 0.1 | 0.2 |  |  |  | 17 | <0 | 0.3 |  |  |
| 4-5 months |  | 1 111 | 9.3 | 15.8 |  |  |  | 1 586 | 1.9 | 25.4 |  |  |
| ≥6 months |  | 5 901 | 49.4 | 84.0 |  |  |  | 4 651 | 5.7 | 74.4 |  |  |
| Missing |  | 4 919 | 41.2 |  |  |  |  | 76 037 | 92.4 |  |  |  |
| **Dairy** |  | N = 8 688 |  |  | 5.9 (1.3) | 6.0 (5.0-6.0) |  | N = 53 813 |  |  | NA^a^ | NA^a^ |
| <6 months |  | 3 473 | 29.1 | 40.0 |  |  |  | 3 412 | 4.1 | 6.3 |  |  |
| 6-8 months |  | 4 931 | 41.3 | 56.8 |  |  |  | 4 073 | 4.9 | 7.6 |  |  |
| ≥9 months |  | 284 | 2.4 | 3.3 |  |  |  | 46 328 | 56.3 | 86.1 |  |  |
| Missing |  | 3 259 | 27.3 |  |  |  |  | 28 478 | 34.6 |  |  |  |
| ^a^Categorical variable in MoBa. ABIS, All Babies in Southeast Sweden study; IQR, interquartile range; MoBa, The Norwegian Mother, Father, and Child Cohort Study. The Latent Class Analysis (LCA) used available data on the food groups above and estimated class membership from available responses, assuming that data were missing at random. | | | | | | | | | | | | |

## Supplementary Table 11. Pooled estimates for the association between the timing of individual food group introduction and inflammatory bowel disease.

| Pooled analysis | Crude analyses | | |  | Adjusted analyses including child’s  sex, birth year, and parental factors^a^ | | |
| --- | --- | --- | --- | --- | --- | --- | --- |
| Characteristic | N | Event n | HR (95% CI) |  | N | Event n | HR (95% CI) |
| Timing of any food introduction | |  |  |  |  |  |  |
| *Before 4 months* | 6 199 | 36 | 1.08 (0.71 1.63) |  | 5 838 | 35 | 1.04 (0.67, 1.60) |
| *4-5 months* | 71 905 | 299 | 0.87 (0.66, 1.14) |  | 68 629 | 282 | 0.83 (0.63, 1.10) |
| *6 months or later* | 14 790 | 64 | (Reference) |  | 14 237 | 63 | (Reference) |
| Food introduction timing by LCA pattern | |  |  |  |  |  |  |
| *Early introducers* | 2 754 | 15 | 1.24 (0.73, 2.12) |  | 2 581 | 14 | 1.24 (0.71, 2.17) |
| *Mid introducers* | 22 973 | 111 | 1.05 (0.82, 1.34) |  | 21 585 | 106 | 1.09 (0.84, 1.41) |
| *Mid-Late introducers* | 46 614 | 165 | (Reference) |  | 44 825 | 155 | (Reference) |
| *Late introducers* | 19 288 | 83 | 1.07 (0.82, 1.40) |  | 18 611 | 81 | 1.12 (0.85, 1.48) |
| Any cereals |  |  |  |  |  |  |  |
| *Before 4 months* | 4 915 | 25 | 1.11 (0.71, 1.75) |  | 4 632 | 24 | 1.11 (0.68, 1.79) |
| *4-5 months* | 67 235 | 263 | 0.91 (0.71, 1.17) |  | 64 199 | 248 | 0.89 (0.69, 1.15) |
| *6 months or later* | 17 210 | 79 | (Reference) |  | 16 606 | 77 | (Reference) |
| Cereals with gluten |  |  |  |  |  |  |  |
| *Before 4 months* | 940 | 7 | 1.45 (0.67, 3.12) |  | 849 | 6 | 1.37 (0.59, 3.16) |
| *4-5 months* | 25 186 | 138 | 1.21 (0.93, 1.57) |  | 23 557 | 127 | 1.21 (0.92, 1.60) |
| *6 months or later* | 26 012 | 96 | (Reference) |  | 25 077 | 93 | (Reference) |
| Fruits or berries |  |  |  |  |  |  |  |
| *Before 4 months* | 1 218 | 8 | 1.24 (0.60, 2.55) |  | 1 169 | 8 | 1.25 (0.61, 2.56) |
| *4-5 months* | 42 363 | 190 | 1.01 (0.79, 1.28) |  | 40 595 | 178 | 0.98 (0.77, 1.27) |
| *6 months or later* | 23 212 | 102 | (Reference) |  | 22 346 | 98 | (Reference) |
| Vegetables or potatoes |  |  |  |  |  |  |  |
| *Before 4 months* | 1 177 | 9 | 1.26 (0.59, 2.67) |  | 1 125 | 9 | 1.28 (0.60, 2.74) |
| *4-5 months* | 38 025 | 180 | 0.97 (0.66, 1.44) |  | 36 176 | 170 | 0.98 (0.63, 1.52) |
| *6 months or later* | 28 146 | 112 | (Reference) |  | 27 012 | 107 | (Reference) |
| Meat |  |  |  |  |  |  |  |
| *Before 4 months* | 196 | 1 | NA^c^ |  | 181 | 1 | NA^c^ |
| *4-5 months* | 16 937 | 95 | 1.11 (0.84, 1.45) |  | 15 916 | 91 | 1.14 (0.86, 1.51) |
| *6 months or later* | 29 780 | 122 | (Reference) |  | 28 655 | 116 | (Reference) |
| Fish |  |  |  |  |  |  |  |
| *Before 4 months* | 33 | 0 | NA |  | 31 | 0 | NA |
| *4-5 months* | 2 697 | 16 | 1.19 (0.39, 3.64) |  | 2 583 | 16 | 1.21 (0.41, 3.64) |
| *6 months or later* | 10 552 | 75 | (Reference) |  | 10 213 | 72 | (Reference) |
| Dairy |  |  |  |  |  |  |  |
| *Before 6 months* | 6 885 | 48 | 0.89 (0.49, 1.60) |  | 6 471 | 45 | 0.88 (0.46, 1.69) |
| *6-8 months* | 9 004 | 77 | (Reference) |  | 8 667 | 75 | (Reference) |
| *9 months or later* | 46 612 | 147 | 0.76 (0.47, 1.24) |  | 44 928 | 143 | 0.75 (0.45, 1.23) |
| ^a^Adjusted for child’s sex and birth year, maternal age and education level, parental inflammatory bowel disease, and origin. ^b^Adjusted for child’s sex and birth year, maternal age and education level, parental inflammatory bowel disease, origin, any breastfeeding duration, and the start of formula feeding. ^c^Unable to pool cohort-specific estimates due to no events in ABIS. ^c^Unable to pool cohort-specific estimates due to no events in ABIS or MoBa. ABIS, All Babies in Southeast Sweden study; CI, Confidence Interval; HR, Hazard Ratio; LCA, Latent Class Analysis; MoBa, The Norwegian Mother, Father, and Child Cohort Study. | | | | | | | |

## Supplementary Table 12. Timing of any complementary food introduction and identified food group patterns and the risk of inflammatory bowel disease in ABIS and MoBa cohorts.

|  | | Crude analyses | | | |  | Adjusted analyses including child’s  sex, birth year, and parental factors^a^ | | |  | Sensitivity analysis adjusted for  infant feeding practices^b^ | | |
| --- | --- | --- | --- | --- | --- | --- | --- | --- | --- | --- | --- | --- | --- |
| Characteristic | Incidence per  100'000 PYR (95% CI) | | N | Event n | HR (95% CI) |  | N | Event n | HR (95% CI) |  | N | Event n | HR (95% CI) |
| ABIS | |  |  |  |  |  |  |  |  |  |  |  |  |
| Timing of any food introduction | | | | | | | | | | | | | |
| *Before 4 months* | | 40.1 (21.4, 68.6) | 1 341 | 13 | 0.93 (0.43, 2.04) |  | 1 265 | 13 | 0.91 (0.41, 2.01) |  | 641 | 7 | 0.79 (0.19, 3.27) |
| *4-5 months* | | 43.3 (35.2, 52.7) | 9 453 | 99 | 1.01 (0.55, 1.83) |  | 9 101 | 95 | 0.99 (0.54, 1.82) |  | 3 424 | 37 | 0.83 (0.25, 2.75) |
| *6 months or later* | | 42.8 (22.1, 74.8) | 1 153 | 12 | (Reference) |  | 1 095 | 12 | (Reference) |  | 236 | 3 | (Reference) |
| Food introduction timing  by LCA pattern | | | | | | | | | | | | | |
| *Early introducers* | | 55.1 (17.9, 128.5) | 375 | 5 | 1.35 (0.52, 3.50) |  | 357 | 5 | 1.47 (0.57, 3.81) |  | 254 | 3 | 1.26 (0.36, 4.42) |
| *Mid introducers* | | 43.9 (31.7, 59.1) | 4 046 | 43 | 1.07 (0.66, 1.74) |  | 3 944 | 42 | 1.13 (0.68, 1.87) |  | 2 024 | 26 | 1.43 (0.68, 3.01) |
| *Mid-Late introducers* | | 40.8 (26.7, 59.8) | 2 611 | 26 | (Reference) |  | 2 542 | 25 | (Reference) |  | 1 099 | 10 | (Reference) |
| *Late introducers* | | 41.7 (26.7, 62.0) | 2 352 | 24 | 1.02 (0.59, 1.78) |  | 2 287 | 23 | 1.00 (0.57, 1.76) |  | 811 | 7 | 0.95 (0.36, 2.51) |
| MoBa | |  |  |  |  |  |  |  |  |  |  |  |  |
| Timing of any food introduction | | | | | | | | | | | | | |
| *Before 4 months* | | 29.7 (18.8, 44.5) | 4 858 | 23 | 1.14 (0.70, 1.86) |  | 4 573 | 22 | 1.09 (0.65, 1.84) |  | 4 040 | 19 | 1.25 (0.66, 2.38) |
| *4-5 months* | | 20.7 (18.0, 23.8) | 62 452 | 200 | 0.84 (0.62, 1.13) |  | 59 528 | 187 | 0.79 (0.58, 1.08) |  | 41 710 | 123 | 0.81 (0.51, 1.31) |
| *6 months or later* | | 24.8 (18.5, 32.5) | 13 637 | 52 | (Reference) |  | 13 142 | 51 | (Reference) |  | 5 452 | 20 | (Reference) |
| Food introduction timing  by LCA pattern | | | | | | | | | | | | | |
| *Early introducers* | | 26.3 (12.6, 48.4) | 2 379 | 10 | 1.20 (0.63, 2.27) |  | 2 224 | 9 | 1.14 (0.57, 2.27) |  | 1 953 | 8 | 1.37 (0.66, 2.87) |
| *Mid introducers* | | 22.6 (17.6, 28.7) | 18 927 | 68 | 1.04 (0.78, 1.39) |  | 17 641 | 64 | 1.08 (0.80, 1.46) |  | 13 165 | 50 | 1.27 (0.89, 1.81) |
| *Mid-Late introducers* | | 20.6 (17.4, 24.4) | 44 003 | 139 | (Reference) |  | 42 283 | 130 | (Reference) |  | 29 393 | 82 | (Reference) |
| *Late introducers* | | 22.6 (17.2, 29.2) | 16 936 | 59 | 1.09 (0.80, 1.47) |  | 16 324 | 58 | 1.16 (0.85, 1.59) |  | 7 262 | 23 | 1.12 (0.70, 1.79) |
| ^a^Adjusted for child’s sex and birth year, maternal age and education level, parental inflammatory bowel disease, and origin. ^b^Adjusted for child’s sex and birth year, maternal age and education level, parental inflammatory bowel disease, origin, any breastfeeding duration, and the start of formula feeding. ABIS, All Babies in Southeast Sweden study; CI, Confidence Interval; HR, Hazard Ratio; LCA, Latent Class Analysis; MoBa, The Norwegian Mother, Father, and Child Cohort Study; PYR, Person Years. | | | | | | | | | | | | | |

## Supplementary Table 13. Timing of any complementary food introduction and identified food group patterns and the risk of Crohn’s disease in ABIS and MoBa cohorts.

|  | | Crude analyses | | | | |  | Adjusted analyses including child’s  sex, birth year, and parental factors^a^ | | |  | Sensitivity analysis adjusted for  infant feeding practices^b^ | | |
| --- | --- | --- | --- | --- | --- | --- | --- | --- | --- | --- | --- | --- | --- | --- |
| Characteristic | Incidence per  100'000 PYR (95% CI) | | | N | Event n | HR (95% CI) |  | N | Event n | HR (95% CI) |  | N | Event n | HR (95% CI) |
| ABIS | |  | |  |  |  |  |  |  |  |  |  |  |  |
| Timing of any food introduction | | | | | | | | | | | | | | |
| *Before 4 months* | | 12.3 (3.4, 31.6) | | 1 341 | 4 | 0.69 (0.18, 2.56) |  | 1 265 | 4 | 0.68 (0.18, 2.59) |  | 641 | 1 | 0.11 (0.01, 1.15) |
| *4-5 months* | | 15.7 (11.0, 21.7) | | 9 453 | 36 | 0.88 (0.34, 2.24) |  | 9 101 | 34 | 0.86 (0.33, 2.25) |  | 3 424 | 14 | 0.33 (0.09, 1.23) |
| *6 months or later* | | 17.8 (5.8, 41.6) | | 1 153 | 5 | (Reference) |  | 1 095 | 5 | (Reference) |  | 236 | 3 | (Reference) |
| Food introduction timing  by LCA pattern | | | | | | | | | | | | | | |
| *Early introducers* | | 22 (2.7, 79.5) | | 375 | 2 | 3.50 (0.64, 19.09) |  | 357 | 2 | 3.63 (0.68, 19.42) |  | 254 | 1 | 1.97 (0.19, 20.50) |
| *Mid introducers* | | 22.4 (14.0, 33.9) | | 4 046 | 22 | **3.56 (1.23, 10.32)** |  | 3 944 | 21 | **3.54 (1.20, 10.41)** |  | 2 024 | 10 | 2.93 (0.62, 13.78) |
| *Mid-Late introducers* | | | 6.3 (1.7, 16.1) | 2 611 | 4 | (Reference) |  | 2 542 | 4 | (Reference) |  | 1 099 | 2 | (Reference) |
| *Late introducers* | | 15.6 (7.1, 29.6) | | 2 352 | 9 | 2.50 (0.77, 8.12) |  | 2 287 | 8 | 2.20 (0.66, 7.28) |  | 811 | 5 | 3.37 (0.64, 17.71) |
| MoBa | |  | |  |  |  |  |  |  |  |  |  |  |  |
| Timing of any food introduction | | | | | | | | | | | | | |  |
| *Before 4 months* | | 15.5 (8.0, 27.0) | | 4 858 | 12 | 1.65 (0.80, 3.39) |  | 4 573 | 12 | 1.66 (0.77, 3.56) |  | 4 040 | 12 | 1.92 (0.81, 4.58) |
| *4-5 months* | | 8.9 (7.1, 11.0) | | 62 452 | 86 | 0.99 (0.60, 1.61) |  | 59 528 | 83 | 0.95 (0.57, 1.57) |  | 41 710 | 61 | 0.92 (0.45, 1.85) |
| *6 months or later* | | 9 (5.4, 14.1) | | 13 637 | 19 | (Reference) |  | 13 142 | 19 | (Reference) |  | 5 452 | 9 | (Reference) |
| Food introduction timing  by LCA pattern | | | | | | | | | | | | | | |
| *Early introducers* | | 15.8 (5.8, 34.4) | | 2 379 | 6 | 1.63 (0.71, 3.76) |  | 2 224 | 6 | 1.66 (0.70, 3.92) |  | 1 953 | 6 | 2.12 (0.88, 5.09) |
| *Mid introducers* | | 9 (5.9, 13.1) | | 18 927 | 27 | 0.93 (0.60, 1.47) |  | 17 641 | 25 | 0.93 (0.58, 1.47) |  | 13 165 | 22 | 1.11 (0.66, 1.85) |
| *Mid-Late introducers* | | | 9.2 (7.1, 11.8) | 44 003 | 62 | (Reference) |  | 42 283 | 61 | (Reference) |  | 29 393 | 43 | (Reference) |
| *Late introducers* | | 8.4 (5.3, 12.8) | | 16 936 | 22 | 0.91 (0.56, 1.47) |  | 16 324 | 22 | 0.94 (0.58, 1.54) |  | 7 262 | 11 | 1.02 (0.52, 2.00) |
| ^a^Adjusted for child’s sex and birth year, maternal age and education level, parental inflammatory bowel disease, and origin. ^b^Adjusted for child’s sex and birth year, maternal age and education level, parental inflammatory bowel disease, origin, any breastfeeding duration, and the start of formula feeding. ABIS, All Babies in Southeast Sweden study; CI, Confidence Interval; HR, Hazard Ratio; LCA, Latent Class Analysis; MoBa, The Norwegian Mother, Father, and Child Cohort Study; PYR, Person Years. | | | | | | | | | | | | | | |

## Supplementary Table 14. Timing of any complementary food introduction and identified patterns and the risk of ulcerative colitis in ABIS and MoBa cohorts.

|  | | | Crude analyses | | | |  | Adjusted analyses including child’s  sex, birth year, and parental factors^a^ | | |  | Sensitivity analysis adjusted for  infant feeding practices^b^ | | |
| --- | --- | --- | --- | --- | --- | --- | --- | --- | --- | --- | --- | --- | --- | --- |
| Characteristic | Incidence per  100'000 PYR (95% CI) | | | N | Event n | HR (95% CI) |  | N | Event n | HR (95% CI) |  | N | Event n | HR (95% CI) |
| ABIS | | |  |  |  |  |  |  |  |  |  |  |  |  |
| Timing of any food introduction | | | | | | | | | | | | | | |
| *Before 4 months* | 21.6 (8.7, 44.5) | | | 1 341 | 7 | 1.21 (0.38, 3.80) |  | 1 265 | 7 | 1.16 (0.37, 3.67) |  | 641 | 5 | NA |
| *4-5 months* | 23.1 (17.3, 30.2) | | | 9 453 | 53 | 1.29 (0.52, 3.23) |  | 9 101 | 51 | 1.27 (0.51, 3.15) |  | 3 424 | 16 | NA |
| *6 months or later* | 17.8 (5.8, 41.5) | | | 1 153 | 5 | (Reference) |  | 1 095 | 5 | (Reference) |  | 236 | 0 | (Reference) |
| Food introduction timing  by LCA pattern | | | | | | | | | | | | | | |
| *Early introducers* | 11.0 (0.3, 61.2) | | | 375 | 1 | 0.35 (0.05, 2.59) |  | 357 | 1 | 0.38 (0.05, 2.91) |  | 254 | 1 | 0.71 (0.09, 5.66) |
| *Mid introducers* | 15.3 (8.5, 25.2) | | | 4 046 | 15 | **0.48 (0.25, 0.94)** |  | 3 944 | 15 | 0.52 (0.26, 1.05) |  | 2 024 | 11 | 0.91 (0.32, 2.56) |
| *Mid-Late introducers* | | 31.4 (19.2, 48.5) | | 2 611 | 20 | (Reference) |  | 2 542 | 19 | (Reference) |  | 1 099 | 6 | (Reference) |
| *Late introducers* | 24.3 (13.3, 40.7) | | | 2 352 | 14 | 0.78 (0.39, 1.54) |  | 2 287 | 14 | 0.79 (0.39, 1.57) |  | 811 | 2 | 0.43 (0.09, 2.20) |
| MoBa |  | | |  |  |  |  |  |  |  |  |  |  |  |
| Timing of any food introduction | | | | | | | | | | | | | | |
| *Before 4 months* | 3.9 (0.8, 11.3) | | | 4 858 | 3 | 0.50 (0.14, 1.73) |  | 4 573 | 3 | 0.57 (0.17, 1.97) |  | 4 040 | 2 | 0.76 (0.14, 4.05) |
| *4-5 months* | 6.2 (4.7, 8.0) | | | 62 452 | 60 | 0.87 (0.50, 1.52) |  | 59 528 | 54 | 0.85 (0.48, 1.52) |  | 41 710 | 35 | 1.25 (0.45, 3.48) |
| *6 months or later* | 7.1 (4.0, 11.8) | | | 13 637 | 15 | (Reference) |  | 13 142 | 14 | (Reference) |  | 5 452 | 4 | (Reference) |
| Food introduction timing  by LCA pattern | | | | | | | | | | | | | | |
| *Early introducers* | 2.6 (0.1, 14.7) | | | 2 379 | 1 | 0.40 (0.06, 2.92) |  | 2 224 | 1 | 0.51 (0.07, 3.73) |  | 1 953 | 1 | 0.70 (0.10, 5.13) |
| *Mid introducers* | 7.0 (4.3, 10.7) | | | 18 927 | 21 | 1.08 (0.63, 1.84) |  | 17 641 | 20 | 1.26 (0.72, 2.19) |  | 13 165 | 12 | 1.11 (0.54, 2.29) |
| *Mid-Late introducers* | | 5.9 (4.2, 8.1) | | 44 003 | 40 | (Reference) |  | 42 283 | 35 | (Reference) |  | 29 393 | 23 | (Reference) |
| *Late introducers* | 6.1 (3.5, 10.0) | | | 16 936 | 16 | 1.02 (0.57, 1.82) |  | 16 324 | 15 | 1.09 (0.60, 1.98) |  | 7 262 | 5 | 0.81 (0.31, 2.12) |
| ^a^Adjusted for child’s sex and birth year, maternal age and education level, parental inflammatory bowel disease, and origin. ^b^Adjusted for child’s sex and birth year, maternal age and education level, parental inflammatory bowel disease, origin, any breastfeeding duration, and the start of formula feeding. ABIS, All Babies in Southeast Sweden study; CI, Confidence Interval; HR, Hazard Ratio; LCA, Latent Class Analysis; MoBa, The Norwegian Mother, Father, and Child Cohort Study; PYR, Person Years. | | | | | | | | | | | | | | |

# Supplementary Figures and Materials.

## Supplementary Figure 1. Data collection description

Description of data collection in the All Babies in Southeast Sweden (ABIS) and the Norwegian Mother, Father, and Child (MoBa) cohorts. GW, gestational week; IBD, inflammatory bowel disease; MBR, Medical Birth Registry; MBRN, Medical Birth Registry of Norway.

## Supplementary Figure 2. Directed Acyclic Graph (DAG)

Directed acyclic graph (DAG) describing the analysis of the timing of complementary food introduction and risk of inflammatory bowel disease (IBD). The DAG was created at www.dagitty.net.

## Supplementary Material 1. Descriptives of the Latent Class Analysis (LCA) and identified patterns of food group introduction performed in the ABIS cohort and applied to the MoBa cohort.

| **Classification of food items to food groups used for Latent Class Analysis.** | | | | | | | | | | |
| --- | --- | --- | --- | --- | --- | --- | --- | --- | --- | --- |
| **Food group** | | **MoBa (questionnaire data at 6 and 18 months)** | | | | **ABIS (food diary data at 0–12 months)** | | | | |
| Any cereal  (with or without gluten) | | Rice porridge, maize porridge; Oatmeal, different types; Wheat porridge, all types, rusk porridge; Wheat flour, rusk, semolina, oats; Iron-enriched wheat flour; Helios baby flour; Millet; Rusks/biscuits/bread | | | | Follow-up formula/gruel with gluten; Follow-up formula/gruel without gluten; Wholegrain Follow-up formula/gruel; Corn – Follow-up formula/gruel; Porridge with gluten (wheat/rye); Oats Porridge; Rice Porridge; Semolina Porridge; Bread with gluten (wheat/rye); Pasta/spaghetti; Rice | | | | |
| Cereal with gluten | | Wheat porridge, all types, rusk porridge; Wheat flour, rusk, semolina, oats; Iron-enriched wheat flour; Helios baby flour; Rusks/biscuits/bread | | | | Follow-up formula/gruel with gluten; Wholegrain Follow-up formula/gruel; Porridge with gluten (wheat/rye); Semolina Porridge; Bread with gluten (wheat/rye); Pasta/spaghetti | | | | |
| Fruits and berries | | Home-made fruit puree; Fruit/berry puree in jar | | | | Fruit, berries | | | | |
| Vegetables and potatoes | | Vegetables; Vegetables and meat; Potato/vegetable puree; Meat and vegetable/potato; Fish and vegetable/potato | | | | Potato; Vegetables | | | | |
| Meat | | Vegetables and meat in a jar; Meat and vegetable/potato (homemade) | | | | Meat | | | | |
| Fish | | Fish and vegetable/potato (homemade) | | | | Fish | | | | |
| Dairy | | *At 6 months:*  Normal sweet milk, any type; Sour milk (yogurt, buttermilk, etc.); Organic milk products (milk, yogurt)  *At 18 months:*  Whole milk (sweet); Low-fat milk normal (sweet); Extra low-fat milk (sweet); Skimmed milk (sweet); Yogurt with active Lactobacillus, all types; Other yogurt; Other types of sour milk | | | | Cheese; Milk (cow); Sour milk/yoghurt | | | | |
| **Bayesian information criterion (BIC) values from the Latent Class Analysis identified patterns of one to 15 classes. A lower BIC value indicates a better fit of the model and the maximum likelihood estimate.** | | | | | | | | | | |
|  | | | | | | | | | | |
| **Food group distribution (%) in the identified patterns** | | | | | | | | | | |
|  | **Class 1** | | | | **Class 2** | | | **Class 3** | | **Class 4** |
| **Any cereal** |  | | | |  | | |  | |  |
| *Before 4 months* | 0 | | | | 95 | | | 1 | | 1 |
| *4-5 months* | 2 | | | | 4 | | | 99 | | 97 |
| *6 months or later* | 98 | | | | 1 | | | 0 | | 2 |
| **Cereal with gluten** |  | | | |  | | |  | |  |
| *Before 4 months* | 0 | | | | 76 | | | 0 | | 0 |
| *4-5 months* | 0 | | | | 19 | | | 80 | | 83 |
| *6 months or later* | 100 | | | | 5 | | | 20 | | 17 |
| **Fruits or berries** |  | | | |  | | |  | |  |
| *Before 4 months* | 1 | | | | 40 | | | 1 | | 4 |
| *4-5 months* | 35 | | | | 49 | | | 54 | | 89 |
| *6 months or later* | 64 | | | | 11 | | | 45 | | 6 |
| **Vegetables or potatoes** |  | | | |  | | |  | |  |
| *Before 4 months* | 1 | | | | 53 | | | 1 | | 9 |
| *4-5 months* | 60 | | | | 42 | | | 83 | | 91 |
| *6 months or later* | 39 | | | | 5 | | | 17 | | 0 |
| **Meat** |  | | | |  | | |  | |  |
| *Before 4 months* | 0 | | | | 8 | | | 0 | | 0 |
| *4-5 months* | 14 | | | | 65 | | | 4 | | 89 |
| *6 months or later* | 86 | | | | 27 | | | 96 | | 1 |
| **Fish** |  | | | |  | | |  | |  |
| *Before 4 months* | 0 | | | | 4 | | | 0 | | 0 |
| *4-5 months* | 2 | | | | 34 | | | 1 | | 30 |
| *6 months or later* | 98 | | | | 62 | | | 99 | | 70 |
| **Dairy^a^** |  | | | |  | | |  | |  |
| *Before 6 months* | 11 | | | | 73 | | | 28 | | 61 |
| *6-8 months* | 81 | | | | 26 | | | 68 | | 38 |
| *9 months or later* | 7 | | | | 1 | | | 4 | | 1 |
| **Description of identified patterns^b^** | | | | | | | | | | |
|  | | | | | | | | | | |
| **Class distributions N (%)** | | | **Late**  **introducers** | **Early**  **introducers** | | | **Mid-Late**  **introducers** | | **Mid**  **introducers** | |
| ABIS | | | 2 352 (25.1) | 375 (4.0) | | | 2 611 (27.8) | | 4 046 (43.1) | |
| MoBa | | | 16 936 (20.6) | 2 379 (2.9) | | | 44 004 (53.5) | | 18 927 (23.0) | |
| ^a^The categorization of dairy differed due to questionnaire formulations in MoBa. ^b^In all identified patterns, fish were mostly introduced ≥6 months. ABIS, All Babies in Southeast Sweden study; MoBa, The Norwegian Mother, Father, and Child Cohort Study. | | | | | | | | | | |
|  | | | | | | | | | | |

## Supplementary Material 2. Covariate data description

Information on parental IBD was retrieved from self-reports at birth in ABIS and the Norwegian Patient Register (NPR) in MoBa. Parental origin was categorized into Sweden or any Other country of birth and reported at birth in ABIS. In MoBa, it was reported at 15 weeks of pregnancy and categorized into Norwegian or Other country. The child’s sex and calendar year of birth were reported in the birth questionnaire in ABIS and retrieved from the Medical Birth Registry of Norway (MBRN) in MoBa. Maternal age at delivery was retrieved from the Swedish National Medical Birth Registry in ABIS and MBRN in MoBa and was categorized into age categories: 15–19, 20–24, 25–29, 30–34, 35–39, and 40–44 years. Maternal education level at birth was reported in the at-birth questionnaire in ABIS and at 15 weeks of pregnancy in MoBa, previously described in detail [12]. Any breastfeeding duration (0–<4 months, 4-<6 months, ≥6 months) was reported in the one-year questionnaire in ABIS and the six-month questionnaire in MoBa. The start of the formula feeding introduction was retrieved from the one-year diary in ABIS and the six-month and 18-month questionnaires in MoBa and categorized into three age groups: Before 1 month, 1–3 months, and 4 months or later.

In sensitivity analyses, we additionally accounted for the following covariates: Maternal smoking during pregnancy (yes/no) [13], diet quality at age one year [14], and antibiotic use by age one year (yes/no) [15], which has been described in detail previously. Delivery mode (cesarean or vaginal), birth weight (limited to 270-6999 grams), and gestational age (limited to 22-45 weeks) were retrieved from MBRN in MoBa and from the birth questionnaire in ABIS.

# REFERENCES

1.	Gilat T, Hacohen D, Lilos P, et al. Childhood factors in ulcerative colitis and Crohn's disease. An international cooperative study. Scand J Gastroenterol. 1987;22(8):1009-24.

2.	Koletzko S, Sherman P, Corey M, et al. Role of infant feeding practices in development of Crohn's disease in childhood. Bmj. 1989;298(6688):1617-8.

3.	Koletzko S, Griffiths A, Corey M, et al. Infant feeding practices and ulcerative colitis in childhood. Bmj. 1991;302(6792):1580-1.

4.	Baron S, Turck D, Leplat C, et al. Environmental risk factors in paediatric inflammatory bowel diseases: a population based case control study. Gut. 2005;54(3):357-63.

5.	Garcia-Larsen V, Ierodiakonou D, Jarrold K, et al. Diet during pregnancy and infancy and risk of allergic or autoimmune disease: A systematic review and meta-analysis. PLoS Med. 2018;15(2):e1002507.

6.	Soriano VX, Ciciulla D, Gell G, et al. Complementary and Allergenic Food Introduction in Infants: An Umbrella Review. Pediatrics. 2023;151(2).

7.	Fantodji C, Rousseau MC, Nicolau B, et al. Early life exposures and risk of inflammatory bowel disease: A nested case-control study in Quebec, Canada. Dig Liver Dis. 2024.

8.	Larsen JH, Andersen S, Perminow G, et al. Higher incidence of paediatric inflammatory bowel disease by increasing latitude in Norway, but stable incidence by age. Acta Paediatr. 2024;113(7):1720-7.

9.	Mouratidou N, Malmborg P, Järås J, et al. Identification of Childhood-Onset Inflammatory Bowel Disease in Swedish Healthcare Registers: A Validation Study. Clin Epidemiol. 2022;14:591-600.

10.	Shrestha S, Olén O, Eriksson C, et al. The use of ICD codes to identify IBD subtypes and phenotypes of the Montreal classification in the Swedish National Patient Register. Scand J Gastroenterol. 2020;55(4):430-5.

11.	Everhov Å H, Frisell T, Osooli M, Brooke HL, Carlsen HK, Modig K, et al. Diagnostic accuracy in the Swedish national patient register: a review including diagnoses in the outpatient register. Eur J Epidemiol. 2025. doi: 10.1007/s10654-025-01221-0.

12.	Sigvardsson I, Størdal K, Östensson M, et al. Childhood Socioeconomic Characteristics and Risk of Inflammatory Bowel Disease: A Scandinavian Birth Cohort Study. Inflamm Bowel Dis. 2023.

13.	Sigvardsson I, Ludvigsson J, Andersson B, et al. Tobacco Smoke Exposure in Early Childhood and Later Risk of Inflammatory Bowel Disease: A Scandinavian Birth Cohort Study. J Crohns Colitis. 2024;18(5):661-70.

14.	Guo A, Ludvigsson J, Brantsæter AL, et al. Early-life diet and risk of inflammatory bowel disease: a pooled study in two Scandinavian birth cohorts. Gut. 2024;73(4):590-600.

15.	Mårild K, Lerchova T, Östensson M, et al. Early-Life Infections, Antibiotics and Later Risk of Childhood and Early Adult-Onset Inflammatory Bowel Disease: Pooled Analysis of Two Scandinavian Birth Cohorts. Aliment Pharmacol Ther. 2024.
